# Single-cell transcriptome delineating asymmetric dynamics of CD4^+^ T and CD8^+^ T cell lineage commitment in human prenatal thymus

**DOI:** 10.3389/fimmu.2026.1823728

**Published:** 2026-05-19

**Authors:** Yang Yang, Yingpeng Yao, Shiga Jibu, Yijun Yin, Zongcheng Li, Yanli Ni, Bing Liu, Yu Lan

**Affiliations:** 1 Key Laboratory for Regenerative Medicine of Ministry of Education, Institute of Hematology, School of Medicine, Jinan University, Guangzhou, China; 2 State Key Laboratory of Experimental Hematology, National Clinical Research Center for Blood Diseases, Haihe Laboratory of Cell Ecosystem, Institute of Hematology & Blood Diseases Hospital, Chinese Academy of Medical Sciences & Peking Union Medical College, Tianjin, China; 3 State Key Laboratory of Experimental Hematology, Haihe Laboratory of Cell Ecosystem, Institute of Hematology, Senior Department of Hematology, Fifth Medical Center of Chinese PLA General Hospital, Beijing, China; 4 Tianjin Institutes of Health Science, Tianjin, China

**Keywords:** CD4/CD8 lineage commitment, human prenatal thymus, selection intermediates, T cell differentiation, thymocyte development

## Abstract

**Introduction:**

The thymus provides a specialized microenvironment for T cell development and selection, yet the cellular heterogeneity and molecular dynamics that govern human prenatal thymopoiesis remain incompletely characterized.

**Methods:**

We constructed an integrative single-cell atlas of human prenatal thymocytes from 7 to 23 post-conception weeks by combining five published datasets. Selection intermediates were classified based on coreceptor expression patterns. Pseudotime analysis, regulon profiling, metabolic analysis, and cell–cell communication modeling were applied to characterize developmental dynamics.

**Results:**

We identified three transitional populations, Sel. int. DP, Sel. int. CD4, and Sel. int. CD8, positioned between DP and single-positive stages. Critically, our findings reveal that CD4/CD8 lineage commitment in the developing human thymus is not a single event, but an asymmetric, multi-stage dynamic process. This asymmetry manifests in three distinct dimensions. First, at the signaling level, Sel. int. CD4 cells exhibit enriched TCR and cytokine signaling activities compared to their Sel. int. CD8 counterparts. Second, at the temporal level, CD4 lineage traits emerge coincident with cellular activation, whereas CD8 lineage characteristics appear only after activation subsides. Third, at the microenvironmental level, Sel. int. CD8 and CD8^+^ T cells display the most extensive interaction networks with thymic stromal cells. Pseudotime analysis delineated two developmental paths branching at the Sel. int. DP stage toward the CD4^+^ and CD8^+^ lineages, revealing four distinct gene expression patterns encompassing activation, viral response, differentiation, and apoptotic programs.

**Discussion:**

Collectively, this atlas provides a comprehensive resource for understanding the asymmetric, multi-stage dynamics of human prenatal T cell development and the cellular crosstalk that orchestrates CD4/CD8 lineage commitment.

## Introduction

T cell development is orchestrated within the unique microenvironment of the thymus. This niche, composed of specialized stromal cells and a complex cytokine milieu, provides the essential signals that guide T cell precursors through sequential stages of specification, commitment, and selection (1). During thymopoiesis, hematopoietic progenitors enter the thymus and differentiate through CD4^-^CD8^-^ double-negative (DN), CD4^+^CD8^+^ double-positive (DP), and finally CD4^+^ or CD8^+^ single-positive (SP) stages (2). This developmental journey is critically dependent on cellular interactions with the thymic microenvironment, which includes cortical thymic epithelial cells (cTECs), medullary thymic epithelial cells (mTECs), dendritic cells (DCs), and fibroblasts (3, 4). The generation of a self-tolerant T cell repertoire (TCR) capable of recognizing foreign antigens is dictated by this complex thymic microenvironment, with mTECs playing a pivotal role through their unique ability to express thousands of tissue-restricted self-antigens (5).

The decision by individual DP thymocytes to terminate synthesis of one coreceptor molecule and differentiate into either CD4^+^ or CD8^+^ T cells, a process known as lineage commitment, has been the subject of intense investigation for decades (5–7). Studies in mouse models have revealed that the intrathymic signals inducing commitment to CD4 versus CD8 lineages are markedly asymmetric (8). During thymic development, thymocytes adjust their TCR response based on the strength of their reactivity to self-peptide major histocompatibility complex (MHC), a tuning process that allows thymocytes with a range of self-reactivities to survive positive selection and contribute to a diverse T cell pool (9, 10). A seminal study using single-cell RNA sequencing to classify mouse thymocyte selection intermediates based on coreceptor gene expression revealed that, in the unperturbed thymus, Cd4^+^Cd8a^-^ selection intermediates appear before Cd4^-^Cd8a^+^ selection intermediates, although the timing of these subsets is flexible according to the strength of TCR signals. This finding suggests that selection intermediates discriminate MHC class prior to the loss of coreceptor expression, with signal strength informing the timing of coreceptor gene activity and ultimately CD4/CD8 lineage choice (11). More recently, single-cell multiomic analysis of thymocyte development has identified sequential waves of TCR signaling that first initiate CD4^+^ T cell lineage differentiation and then CD8^+^ T cell lineage specification (12). The “sequential selection” model proposed by Baldwin and Robey posits that MHC specificity influences lineage choice through differential signal strengths and kinetics, with thymocytes undergoing a stepwise process of activation and commitment (5, 12, 13). A more recent study further reveals that the functional diversity of CD8^+^ T cells is predetermined in the thymus, where distinct MHC-I-bound self-peptides actively select for specific CD8^+^ T cell lineages with differing functional potentials (11). Despite these advances in mouse models, whether the cellular and molecular mechanisms governing CD4/CD8 lineage choice are conserved in human thymocyte development remains largely unknown.

Recent advances in cutting-edge single-cell technologies have enabled unprecedented resolution of thymic cellular heterogeneity. Through the application of single-cell RNA sequencing (scRNA-seq) and single-cell omics technologies, the cellular heterogeneity of thymic cell populations and molecular mechanisms underlying human T cell development, in both the developing fetus and postnatal thymus, have been largely characterized (15–19). Subsequent spatially resolved analysis has mapped thymocyte trajectories to continuous tissue axes, revealing the establishment of the lobular cytokine network and thymic epithelial cell distributions by the second trimester of fetal development (20). The integration of multimodal data from single-cell and spatial transcriptomics has enabled the reconstruction of thymic spatial architecture at single-cell resolution, recapitulating classical cell types and their essential colocalization for T cell development, while also identifying previously unknown colocalization relationships (20). Notably, spatial mapping study has identified divergence in the timing of medullary entry between CD4 and CD8 T cell lineages, with CD8-committed T cells lingering longer in the cortex (20). While these studies have provided valuable insights into human thymic cellular composition and spatial organization, a systematic dissection of the selection intermediates along the CD4 versus CD8 developmental trajectories and the molecular mechanisms underlying T cell fate choice in the human prenatal thymus remains lacking. Moreover, the cellular interactions between developing thymocytes and the thymic microenvironment that orchestrate lineage commitment have not been systematically characterized at single-cell resolution.

We have previously revealed divergent molecular events underlying initial T cell commitment in human thymus (21). In the present study, we constructed an integrative single-cell atlas of human prenatal thymocytes spanning 7 to 23 post-conception weeks by combining published datasets (15, 16, 22–24). Through trajectory inference, regulon analysis, metabolic pathway profiling, and cell-cell communication modeling, we delineated the transcriptional hallmarks, signaling dynamics, and microenvironmental interactions that govern CD4/CD8 lineage choice in the developing human thymus. Our findings extend previous observations from mouse models by providing detailed characterization of selection intermediates in human T cell development, revealing both conserved and species-specific features of T cell lineage commitment.

## Results

### An integrative single-cell atlas of human prenatal thymocytes

**Figure 1:**
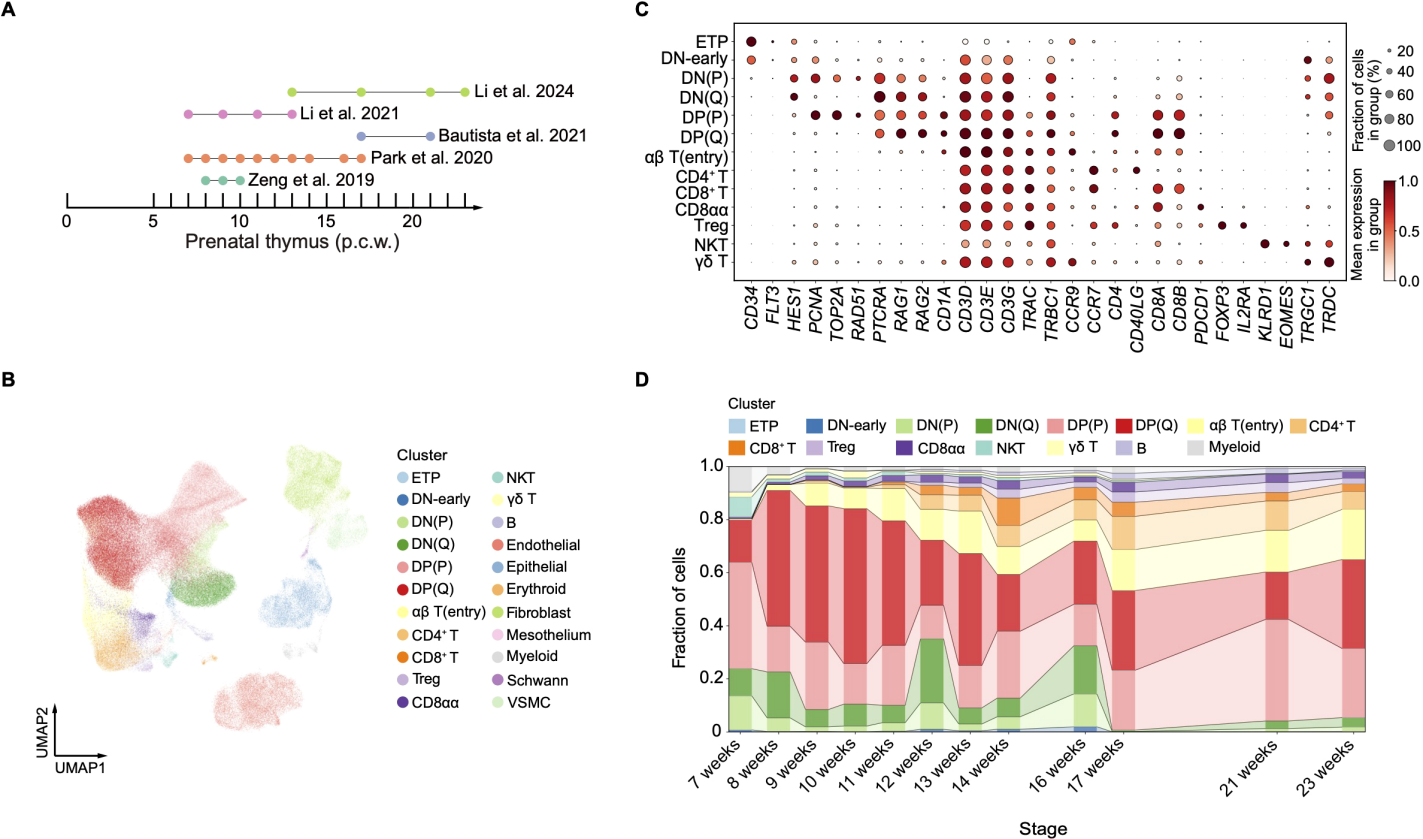
Integrative analysis of human prenatal thymocytes. **(A)** Summary of gestational stage of samples from five published datasets. Each symbol denotes an individual time point. **(B)** Visualization of uniform manifold approximation and projection (UMAP) for cellular composition of human prenatal thymus colored by cell type. **(C)** Dot plot showing feature genes expression in indicated cell clusters. **(D)** Bar graph showing the proportion representation of cell states.

To understand the early stages of human thymus development in a time course manner, we constructed an integrative single-cell atlas of human prenatal thymocytes by combining published available datasets with distinct cell sorting strategies (15, 16, 22–24). The five datasets collectively span human prenatal thymic development from 7 to 23 post-conception weeks (PCW), with variable temporal coverage: Zeng et al. (2019) contributed samples at 8–10 PCW, Park et al. (2020) at 7–17 PCW, Bautista et al. (2021) at 17 and 21 PCW, Li et al. (2021) at 7–13 PCW, and Li et al. (2024) at 13–23 PCW (**Figure 1A**). This temporal continuum allows us to capture both early (first trimester) and mid-to-late (second trimester) stages of thymopoiesis. Following strict quality control (**Supplementary Figure 1A**), the integrated dataset comprised 216,463 cells. After batch effect correction and unsupervised clustering, we identified 22 major cell types, including 16 hematopoietic clusters and 6 non-hematopoietic clusters (**Figure 1B**; **Supplementary Figures 1B–E**). Cell identities were assigned based on the expression profiles of population-specific marker genes (**Figure 1C**; **Supplementary Figure 1D**). Withing T cell development trajectories, we identified early T cell progenitor (ETP), DN, DP, CD4^+^ T and CD8^+^ T cells. Additionally, CD8αα (*CD8A*, *PDCD1*), Treg (*FOXP3*), NKT (*KLRD1*, *EOMES*), and γδ T cells (*TRGC1*, *TRDC*) were also identified. Among non-hematopoietic subsets, endothelial cells (*PECAM1*, *CDH5*), epithelial cells (*KRT19*, *FOXN1*), fibroblast (*COL1A1*, *DCN*), mesothelium (*PRG4*, *MSLN*), Schwann (*MPZ*, *PMP22*), and vascular smooth muscle cells (VSMC; *ACTA2*, *RGS5*) were defined. Next, we examined the developmental kinetics of hematopoietic clusters across embryonic stages (**Figure 1D**; **Supplementary Figure 1G**). NKT cells appeared as early as 7 PCW, with their proportion declining dramatically thereafter (**Figure 1D**). Similarly, γδ T cells emerged at 7 PCW, peaked at 10 PCW, and subsequently decreased (**Figure 1D**). CD8αα T cells were detectable at 7 PCW and then remained at a relatively stable level. CD4^+^ and CD8^+^ T cells arose at 11 PCW, later than αβ T(entry), which was observed at 9 PCW (**Figure 1D**). Between 16 and 17 PCW, DN cells declined sharply, while DP cells and their descendants became the predominant populations in the thymus (**Figure 1D**). In summary, this comprehensive atlas enables detailed characterization of thymic T cell heterogeneity and their dynamic changes during human embryonic development at single-cell resolution.

### Classification of selection intermediates in human prenatal thymocytes

**Figure 2:**
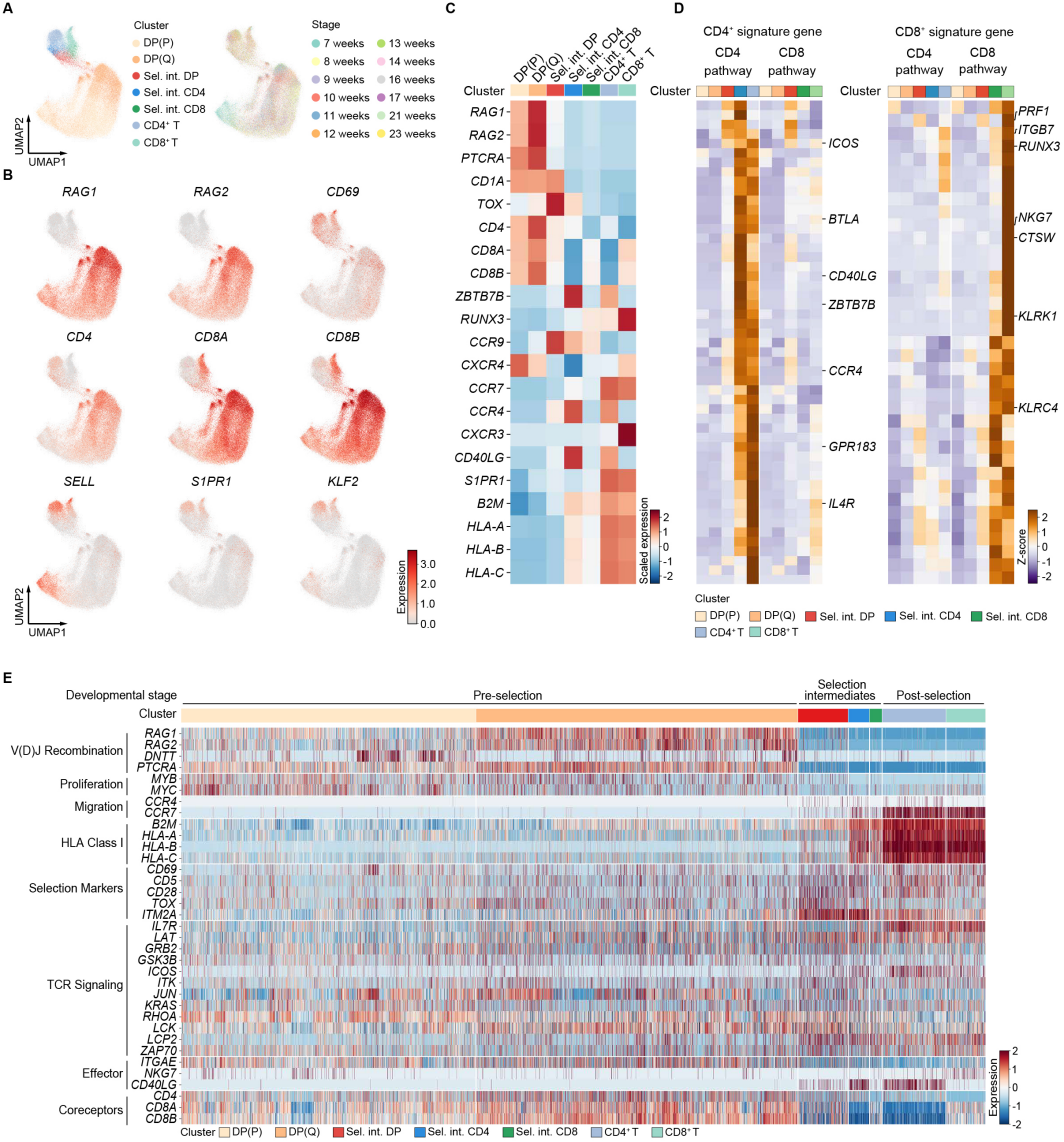
Classification of selection intermediates based on *CD4* and *CD8A* coreceptor gene expression. **(A)** Visualization of UMAP for cellular composition of human prenatal thymus colored by cell type (left) and by gestational stage (right). Sel. int. stands for Selection intermediates. **(B)** UMAP plot showing *RAG1*, *RAG2*, *CD69*, *CD4*, *CD8A*, and *CD8B* gene expression. **(C)** Heatmap showing feature genes expression in indicated cell clusters. **(D)** Heatmaps showing CD4^+^-lineage (left) and CD8^+^-lineage (right) signature gene expression. **(E)** Heatmap showing genes expression across distinct developmental stages (pre-selection, selection intermediates, and post-selection).

To investigate CD4/CD8 lineage commitment in human prenatal thymus, we reconstructed the developmental trajectory from DP cells through αβ T(entry) to single-positive cells (**Supplementary Figure 2A**). We then sought to define selection intermediates within the αβ T(entry) cluster based on coreceptor transcripts expression, as previously described in mice (11). We observed three expression patterns of coreceptor transcripts in αβ T(entry) cluster: cells expressing only *CD4*, only *CD8A*, or co-expressing both *CD4* and *CD8A* (**Supplementary Figure 2B**), suggesting the possibility of classifying selection intermediates among these αβ T (entry) cells. Accordingly, we performed unsupervised clustering on the above-mentioned clusters spanning from 7 to 23 PCW, and divided them into seven refined subpopulations (**Figure 2A**; **Supplementary Figures 2C–E**), including proliferating DP (P), quiescent DP (Q), CD4^+^ T, CD8^+^ T, and three selection intermediates (namely Sel. int. DP, Sel. int. CD4, and Sel. int. CD8). DP (P) and DP (Q) showed high expression of *RAG1* and *RAG2*, consistent with a pre−selection identity (**Figures 2B, C**). In contrast, Sel. int. DP, Sel. int. CD4, and Sel. int. CD8 ceased expression of *RAG1*, *RAG2*, upregulated TCR signaling targets *CD69* and *TOX*, lacked expression of mature markers (*SELL*, *S1PR1*, *KLF2*), and showed lower expression of human leukocyte antigen (HLA) genes (*B2M*, *HLA-A*, *HLA-B*, *HLA-C*) compared to single-positive T cells, implying they had undergone selection (**Figures 2B, C**). Correspondingly, scores for a TCR signaling signature (25) were elevated in all selection intermediates, with Sel. int. CD4 cluster exhibiting higher TCR signaling than Sel. int. CD8 cluster (**Supplementary Figure 2F**). Signature scores for CD4^+^ and CD8^+^ lineages were specifically enriched in the respective mature T cell clusters (**Supplementary Figure 2F**). Notably, elevated CD4^+^ signature (25) scores but not CD8^+^ signature (25) scores were detected as early as in Sel. int. DP cluster and were higher in Sel. int. CD4 than in Sel. int. CD8 cells (**Supplementary Figure 2F**). We then established CD4^+^ and CD8^+^ lineage signatures by identifying gene sets more highly expressed in each lineage compared both to the other lineage and to pre−selection DP cells (**Supplementary Table 2**). Most CD8^+^ signature genes were highly expressed in mature CD8^+^ T cells but showed lower expression at earlier stages, whereas the CD4^+^ signature appeared earlier and displayed varied expression patterns across clusters (**Figure 2D**). We then intended to identify transcriptional programs in each selection intermediates according to coreceptor status, and found TCR signaling genes, TCR signaling targets, and lineage-specific genes showed distinct kinetic patterns (**Figure 2E**). In summary, based on CD4 and CD8 coreceptor gene expression, selection intermediates were classified in human prenatal thymocytes.

### Characterization of transcriptional differences among selection intermediates

**Figure 3:**
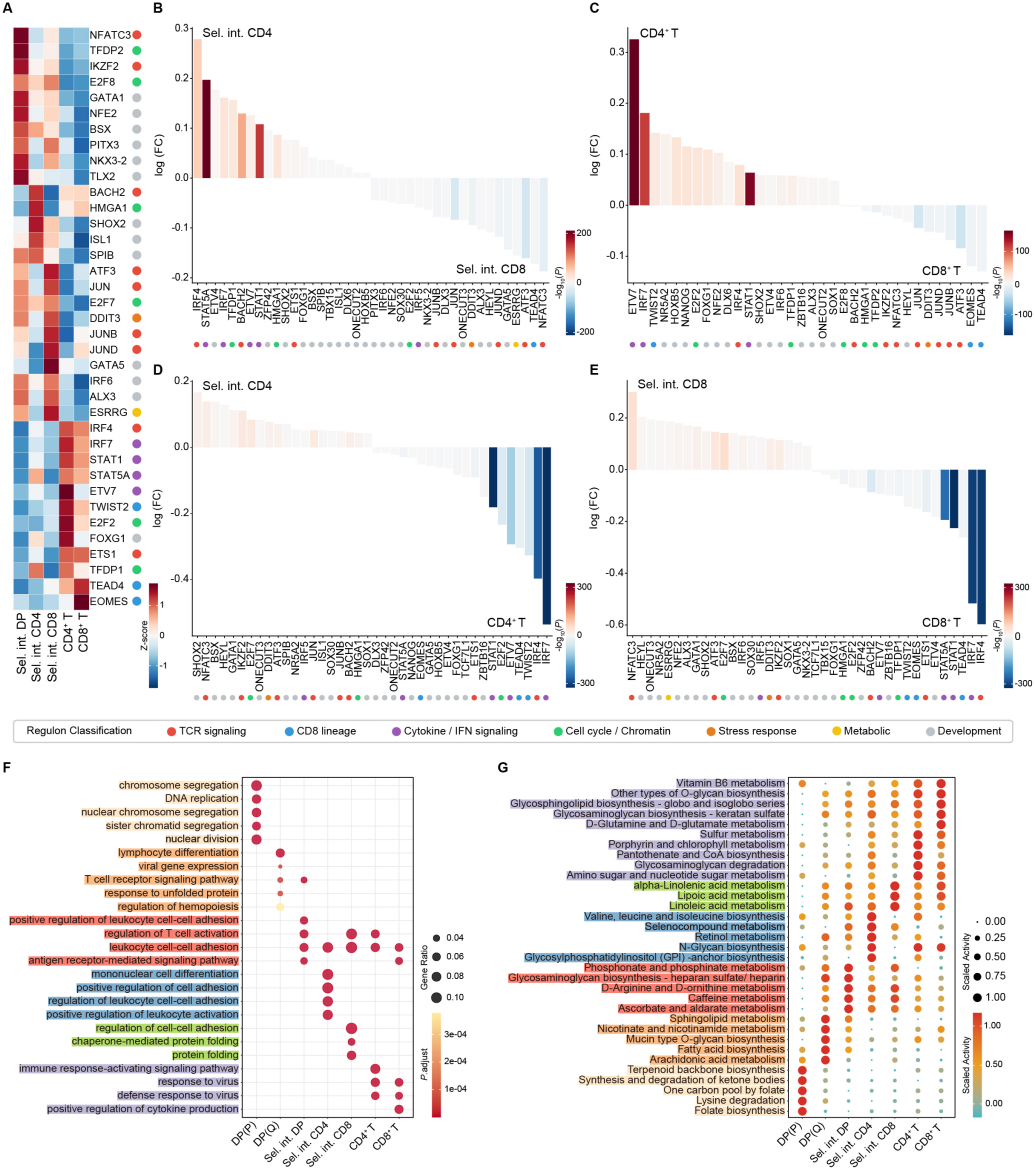
Illustrating transcriptional characteristics of selection intermediates. **(A)** Heatmap showing the enriched regulons in selection intermediates and single-positive cells. Representative regulons are shown on the right. The function of individual regulon is indicated by dot with different color. **(B)** Differentially enriched regulons activities in Sel. int. CD4 versus Sel. int. CD8 cluster. The function of individual regulon is indicated by dot with different color. **(C)** Differentially enriched regulons activities in CD4^+^ T versus CD8^+^ T cluster. The function of individual regulon is indicated by dot with different color. **(D)** Differentially enriched regulons activities in Sel. int. CD4 versus CD4^+^ T cluster. The function of individual regulon is indicated by dot with different color. **(E)** Differentially enriched regulons activities in Sel. int. CD8 versus CD8^+^ T cluster. The function of individual regulon is indicated by dot with different color. **(F)** Dot plot showing top five enriched Gene Ontology (GO) terms for DEGs of indicated cell clusters. Enriched GO terms for each cell subpopulation were color-coded. **(G)** Dot plot showing enrichment of metabolic pathways in indicated cell clusters. Enriched GO terms for each cell subpopulation were color-coded.

To illustrate the transcriptional differences among selection intermediates and mature single-positive cells, we applied single-cell regulatory network inference and clustering (SCENIC) to identify differentially activated transcription factors (TFs) and their regulons in these clusters (**Figure 3A**; **Supplementary Figure 3**). Regulons activity in the three selection intermediates was stage-specific. The Sel. int. DP showed enriched regulons activity related to cell proliferation and cell cycle (NFATC3, TFDP2, E2F8). A regulon associated with CD4^+^ T cell differentiation (BACH2) was highly enriched in the Sel. int. CD4 cluster (26). In contrast, the Sel. int. CD8 cluster showed significant enrichment of AP-1 family regulons (JUN, JUNB, JUND), which play vital roles in T cell differentiation, activation, and proliferation (27–29). The majority of regulon activities were shared between CD4^+^ and CD8^+^ T cells. However, CD4^+^ T cells exhibited higher activity of regulons related to skewing CD8^+^ T cell exhaustion (ETV7) (30). By contrast, EOMES, a master regulator of CD8^+^ T cells (31), was more enriched in CD8^+^ T cluster. Compared with Sel. int. CD8 cluster, the Sel. int. CD4 cluster upregulated regulon activities associated with T cell fate (IRF4, STAT5A) (32, 33) and downregulated regulons activities related with cell proliferation and differentiation (NFATC3, ATF3, and TEAD4) (**Figure 3B**). The differential regulon activities observed between CD4^+^ T and CD8^+^ T cells largely overlapped with those between Sel. int. CD4 and Sel. int. CD8 clusters (**Figure 3C**). In contrast to Sel. int. CD4 cluster, the CD4^+^ T cluster upregulated IRF family regulons (IRF4, IRF7) (**Figure 3D**). Similarly, CD8^+^ T cluster also exhibited enhanced IRF4 and IRF7 activities (**Figure 3E**).

Gene Ontology (GO) term analysis further revealed distinct enrichment patterns among selection intermediates (**Figure 3F**). Pre-selection DP cells exhibited higher enrichment of GO terms related to DNA replication and lymphocyte differentiation (**Figure 3F**). By contrast, the Sel. int. DP cluster was enriched for terms associated with T cell receptor signaling pathway, positive regulation of leukocyte cell-cell adhesion, and antigen receptor-mediated signaling pathway (**Figure 3F**). Transcripts for Sel. int. CD4 cluster were enriched for GO terms linked to leukocyte activation and cell adhesion (**Figure 3F**), a critical step for single-positive thymocytes to recognize self-antigen peptides presented by mTECs (34), suggesting that Sel. int. CD4 cells are poised for negative selection. In contrast, the Sel. int. CD8 cluster exhibited enrichment for terms related to T cell activation and protein folding (**Figure 3F**). Mature CD4^+^ T and CD8^+^ T clusters shared enrichment for GO terms associated with leukocyte cell-cell adhesion and response to virus (**Figure 3F**).

We and others have previously reported that thymocytes at different developmental stages exhibit distinct metabolic patterns (21, 35, 36). We next sought to elucidate the transcriptional differences among selection intermediates from a metabolic perspective (**Figure 3G**). The DP (P) cluster displayed highly active metabolic programs related to acetyl-CoA metabolic network (including terpenoids biosynthesis, ketone body metabolism, and lysine degradation), consistent with their rapid proliferation status (37) (**Figure 3G**). The DP (Q) cluster showed higher activity in lipid-related metabolic pathways (fatty acid biosynthesis, sphingolipid metabolism, arachidonic acid metabolism) as well as nicotinate and nicotinamide metabolism, which are linked to cell membrane synthesis and energy metabolism remodeling (38–40) (**Figure 3G**). Among these selection intermediates, the Sel. int. DP cluster was enriched for pathways including glycosaminoglycan biosynthesis-heparan sulfate/heparin, which servers as coreceptors for fibroblast growth factor and chemokine (41, 42), as well as ascorbate and aldarate metabolism, which promotes T cell maturation (43) (**Figure 3G**). Pathways related to providing cofactors or structural components (branched-chain amino acids, selenocysteine, retinol) for post-translational modification and protein maturation were enriched in the Sel. int. CD4 cluster (**Figure 3G**), suggesting active protein biosynthesis and modification in this cluster. Conversely, the Sel. int. CD8 cluster was more enriched for fatty acid and derivative metabolism (**Figure 3G**), which exert vital roles in T cell responses against tumor and inflammation (44–46). The enriched metabolic pathways in CD4^+^ and CD8^+^ T cells were largely overlapping, encompassing cofactor and energy metabolism, noncanonical amino acid metabolism, and glycan and glycosphingolipid metabolic network (**Figure 3G**).

### CD4^+^ T cell lineage characteristics are initiated early

**Figure 4:**
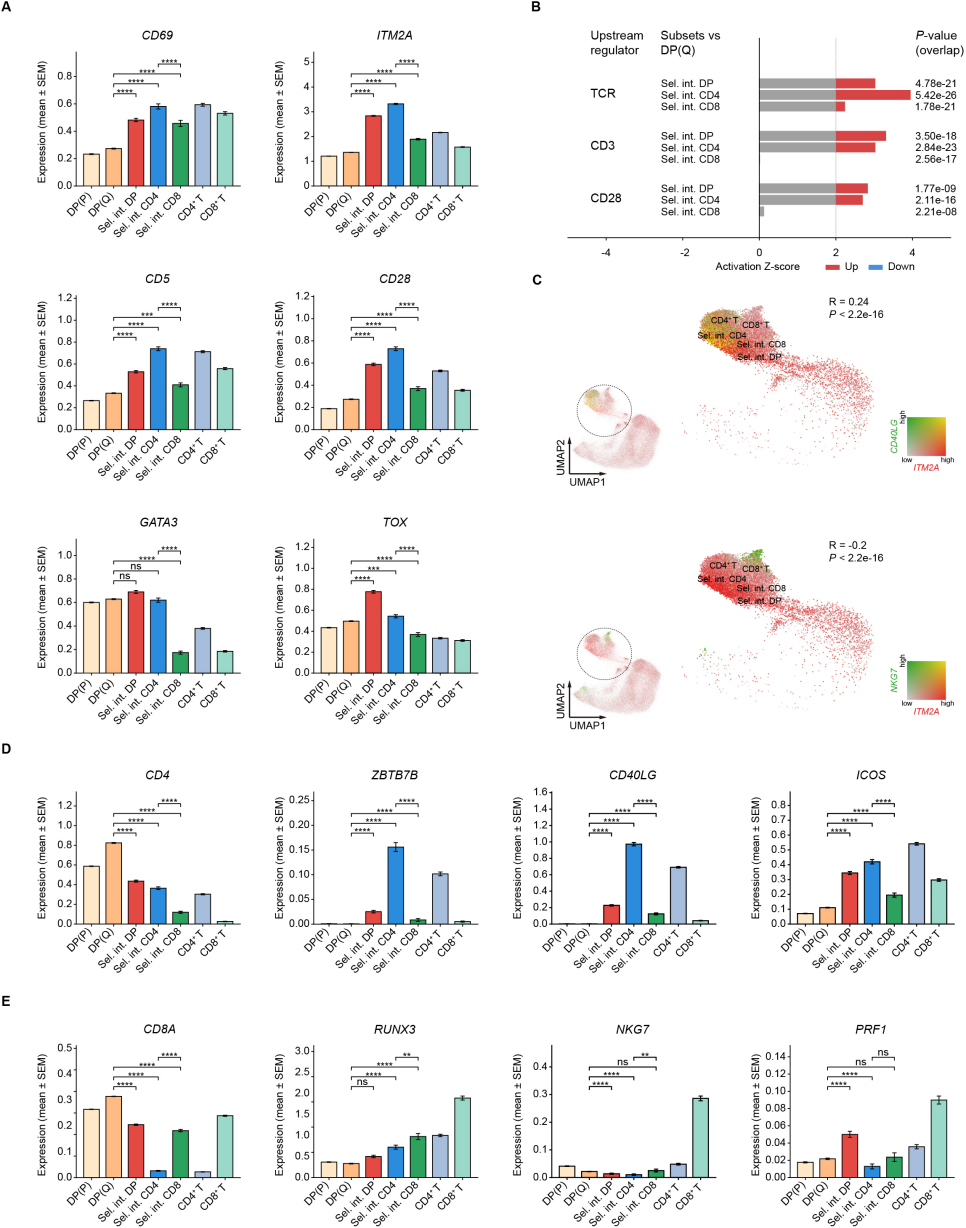
Establishment of CD4 and CD8 lineage characteristics is temporally uncoupled during CD4/CD8 lineage choice. **(A)** Expression of TCR activation genes *CD69*, *ITM2A*, *CD5*, *CD28*, *GATA3*, and *TOX* by selection intermediates. **(B)** IPA analysis of pathway activity downstream of the TCR, CD3 and CD28. Activation Z-scores above 2 and below -2 are considered significant. **(C)** Co-expression of the activation marker *ITM2A* with the CD4 lineage marker *CD40LG* (top panel) and CD8 lineage marker *NKG7* (bottom panel). The enlarged views of the area enclosed by the dotted circle are shown on the right. **(D)** Expression of CD4 lineage genes *CD4*, *ZBTB7B*, *CD40LG*, and *ICOS* by selection intermediates. **(E)** Expression of CD8 lineage genes *CD8A*, *RUNX3*, *NKG7*, and *PRF1* by selection intermediates.

Next, we examined the expression of genes associated with TCR signaling, cytokine signaling, and lineage specification in selection intermediates, all of which are critical for T cell lineage commitment. The expression of TCR-driven activation genes, including *CD69*, *ITM2A*, *CD5* and *CD28*, was significantly higher in selection intermediates than in pre-selection DP cells (**Figure 4A**). Notably, Sel. int. CD4 expressed these TCR-related genes at higher levels than Sel. int. CD8 (**Figure 4A**). Ingenuity Pathway Analysis (IPA) further suggested activation of downstream TCR pathway in both Sel. int. CD4 and Sel. int. CD8; however, activation of pathways downstream of CD3 and of CD28 was observed exclusively in Sel. int. CD4 (**Figure 4B**). These results indicate more robust TCR signaling activity in Sel. int. CD4 compared to Sel. int. CD8.

Expression of *IL7R* gene was modestly elevated in Sel. int. CD4 but not in Sel. int. CD8 relative to pre-selection DP (**Supplementary Figure 4A**). Accordingly, expression of the IL7R signaling target gene *GIMAP4* was also significantly elevated in Sel. int. CD4 (**Supplementary Figure 4A**). In contrast, expression of *SOCS1*, which encodes the suppressor of cytokine signaling, was markedly downregulated in both Sel. int. CD4 and Sel. int. CD8 compared to pre-selection DP (**Supplementary Figure 4A**). Moreover, expression of the PKC-NFκB signaling genes *REL* and *RELB* was significantly upregulated in Sel. int. CD4 (**Supplementary Figure 4A**). IPA analysis further revealed that members of TNF, JAK, interleukin, and STAT families were activated in Sel. int. CD4 but not in Sel. int. CD8. Although significant activation downstream of IFNG and IL27 was observed in both Sel. int. CD4 and Sel. int. CD8, the activation scores were substantially higher in Sel. int. CD4 (**Supplementary Figure 4B**). These results suggest that cytokine signaling pathway activity is potentially preferentially enriched in Sel. int. CD4.

To delineate the temporal relationship between thymocyte activation and lineage commitment, we analyzed the dynamic expression of CD4 and CD8 lineage genes. A significant positive correlation was observed between expression of the CD4 lineage gene *CD40LG* and the activation marker *ITM2A*, with frequent co-expression of these genes in individual cells (**Figure 4C**). By contrast, upregulation of the CD8 lineage marker *NKG7* occurred only after expression of the activation marker *ITM2A* had subsided, resulting in a negative correlation between them (**Figure 4C**). CD4 lineage genes, including *ZBTB7B*, *CD40LG*, and *ICOS*, were expressed by Sel. int. CD4 (**Figure 4D**), whereas expression of CD8 lineage genes such as *RUNX3*, *NKG7*, and *PRF1* was largely restricted to CD8^+^ T cells (**Figure 4E**). These results are also consistent with those observed in **Figure 2D**. Collectively, these findings indicate that during CD4/CD8 lineage choice, the expression of CD4 lineage traits coincided with cellular activation, while the emergence of CD8 lineage characteristics follows it.

### Dynamic molecular characteristics during CD4^+^ and CD8^+^ T cell lineage commitment

**Figure 5:**
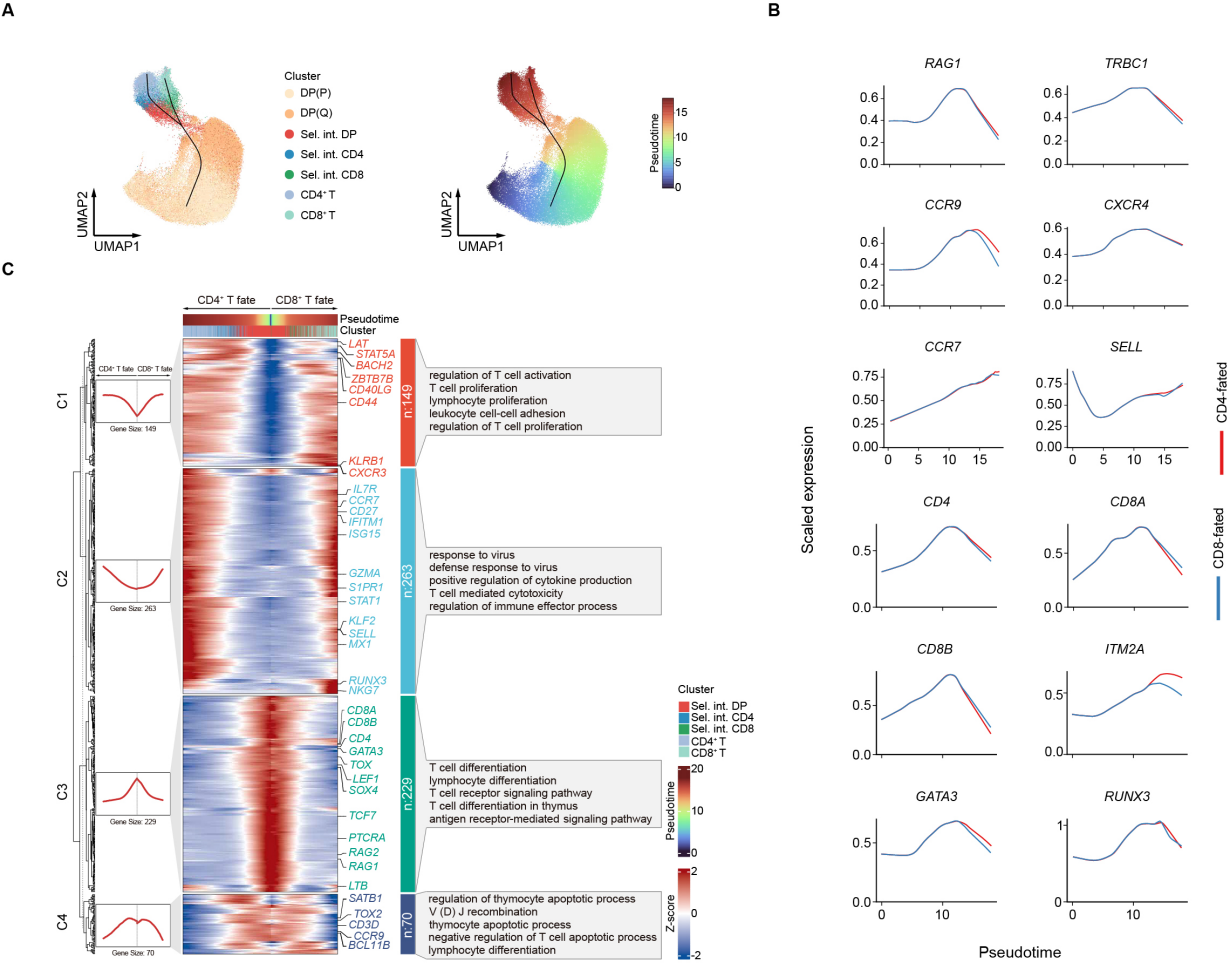
Dynamic molecular programs during CD4^+^ and CD8^+^ T cell lineage commitment. **(A)** Left: Developmental trajectory of thymocytes inferred by slingshot algorithm. Right: UMAP plot of thymocytes with cells colored by Slingshot pseudotime. **(B)** Expression over pseudotime as in **A** of key factors. **(C)** Heatmap showing the expression changes of pattern genes along the trajectory of CD4^+^ and CD8^+^ T cell lineage commitment.

To further delineate CD4^+^ and CD8^+^ T cell lineage commitment across the developmental trajectory, pseudotime inference was performed using Slingshot (**Figure 5A**). Two developmental paths were identified, both originating from DP (P), progressing through DP (Q), branching at the Sel. int. DP stage, and subsequently diverging into CD4^+^ and CD8^+^ lineage cells. This result was corroborated by RNA velocity and Monocle 3 analysis (**Supplementary Figure 5**). The inferred pseudotime recapitulated the temporal order of known expression dynamics (12, 47), including early downregulation of TCR recombination marker *RAG1*, successive downregulation of early marker *CCR9*, and late upregulation of the maturation marker *SELL* (**Figure 5B**). For CD4^+^ T lineage, *ITM2A* was consistently upregulated across the developmental trajectory compared to CD8^+^ T lineage (**Figure 5B**). Lineage-specific genes exhibited higher expression in their corresponding lineages (**Figure 5B**). Based on dynamically expressed genes along the developmental trajectories, we identified four distinct gene expression patterns (**Figure 5C**). Genes in pattern 1 were sharply upregulated along the trajectory of CD4^+^ and CD8^+^ lineage divergence and were associated with T cell activation and proliferation (*STAT5A*, *CD44*) (48). Genes in pattern 2 were gradually upregulated along both T cell lineages and were enriched for functions related to response to virus (*GZMA*, *NKG7*) and regulation of immune effector process (*RUNX3*, *STAT1*, *ISG15*). Pattern 3 comprised genes related to T cell differentiation (*CD4*, *CD8A*, *CD8B*, *GATA3*, *TOX*, *TCF7*) and T cell receptor signaling pathway (*PTCRA*, *RAG1*, *RAG2*), which were sharply declined over the course of development. Genes associated with apoptotic process (*TOX2*, *SATB1*) (49, 50) were highly enriched in pattern 4 and exhibited gradual downregulation during T cell fate determination. Collectively, these findings provide a comprehensive landscape of activation and repression of genetic programs during CD4^+^ and CD8^+^ T cell lineage commitment.

### Cellular interactions between thymocytes and stromal cells during T cell lineage choice

**Figure 6:**
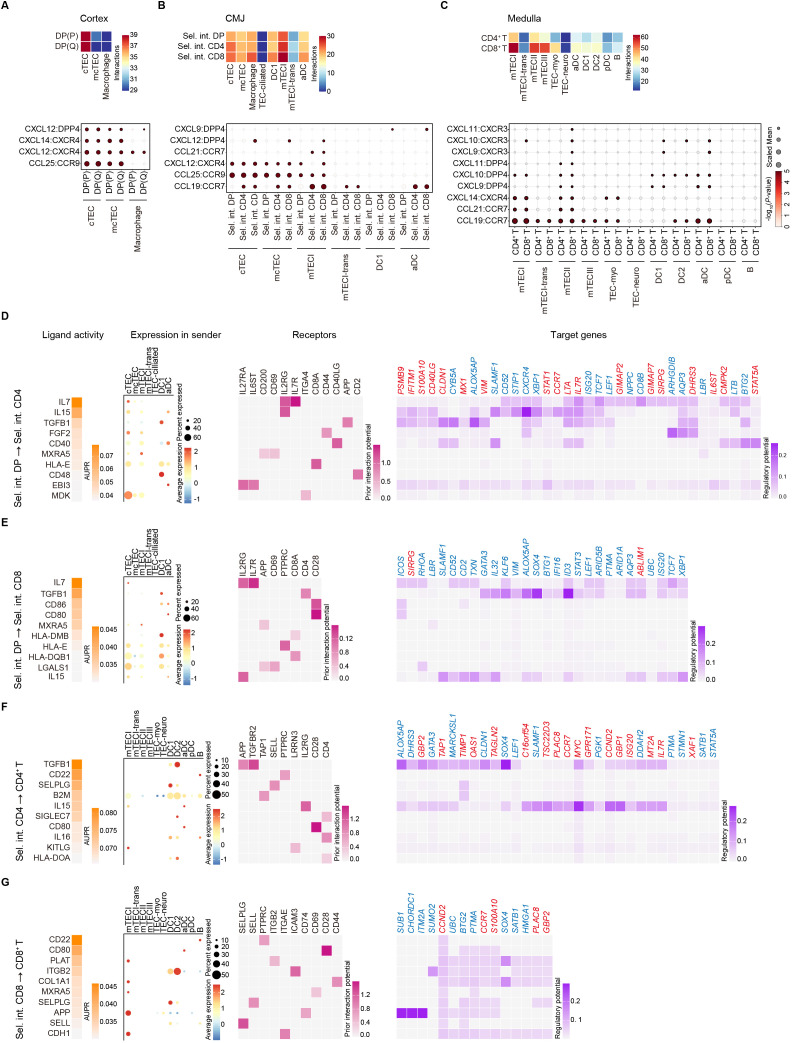
Thymocyte-stromal cell crosstalk during T cell lineage commitment. **(A)** Top panel: Heatmap showing interactions between DP cells and stromal cells. Bottom panel: Dot plot depicting chemokine interactions between DP cells and stromal cells. **(B)** Top panel: Heatmap showing interactions between selection intermediates and stromal cells. Bottom panel: Dot plot depicting chemokine interactions between selection intermediates and stromal cells. **(C)** Top panel: Heatmap showing interactions between single-positive cells and stromal cells. Bottom panel: Dot plot depicting chemokine interactions between single-positive cells and stromal cells. **(D)** NicheNet analysis of ligand-receptor interactions from Sel. int. DP to Sel. int. CD4. Far left: The ligand activity is shown. Left: The percentage of cells per cell type expressing each ligand on average is shown. Right: Receptors with the highest interaction potential with the prioritized ligands are depicted and hierarchically clustered. Far right: Heatmap displaying the target gene candidates. **(E)** NicheNet analysis of ligand-receptor interactions from Sel. int. DP to Sel. int. CD8. Far left: The ligand activity is shown. Left: The percentage of cells per cell type expressing each ligand on average is shown. Right: Receptors with the highest interaction potential with the prioritized ligands are depicted and hierarchically clustered. Far right: Heatmap displaying the target gene candidates. **(F)** NicheNet analysis of ligand-receptor interactions from Sel. int. CD4 to CD4^+^ T. Far left: The ligand activity is shown. Left: The percentage of cells per cell type expressing each ligand on average is shown. Right: Receptors with the highest interaction potential with the prioritized ligands are depicted and hierarchically clustered. Far right: Heatmap displaying the target gene candidates. **(G)** NicheNet analysis of ligand-receptor interactions from Sel. int. CD8 to CD8^+^ T. Far left: The ligand activity is shown. Left: The percentage of cells per cell type expressing each ligand on average is shown. Right: Receptors with the highest interaction potential with the prioritized ligands are depicted and hierarchically clustered. Far right: Heatmap displaying the target gene candidates.

The production of a functional T cell pool in the thymus is largely controlled by TECs, which provide essential guidance, resources, and signals for migration, growth, specialization, and maintenance that support the development and maturation of thymocytes (51). To investigate the cellular interactions between thymocytes and stromal cells during T cell lineage choice, we first retrieved myeloid and epithelial clusters and re-clustered them into several subpopulations (**Figure 1B**, **Supplementary Figures 6A–F**). We then performed cell-cell communication analysis using CellPhoneDB to systematically interrogate ligand-receptor pairs across various cell types. In the cortex, prominent interactions between cTEC and DP cells were observed, primarily mediated by CCL25−CCR9 and CXCL12−CXCR4, which promote homing and retention of cells in the thymus (52, 53) (**Figure 6A**). In the corticomedullary junction (CMJ) region, mTECI showed the strongest interactions with selection intermediates mainly through CCL25−CCR9 and CCL19−CCR7 (except Sel. int. DP) (**Figure 6B**), which is vital for migration of thymocytes into the thymic medulla (54). Additionally, selection intermediates also showed interactions with cTEC, mcTEC, macrophage, DCs (**Figure 6B**). Notably, Sel. int. CD8 cells displayed stronger interactions with stromal cells compared to the other selection intermediates (**Figure 6B**). Within the medulla, single positive cells showed the strongest interactions with mTECI relative to mTECII and mTECIII through CCL19−CCR7, CCL21−CCR7, and CXCL14−CXCR4 (**Figure 6C**). Moreover, CD8^+^ T cells exhibited more extensive interactions with these stromal cells than CD4^+^ T cells (**Figure 6C**).

We next applied NicheNet analysis (55) to infer ligand-receptor interactions potentially driving gene expression changes during T cell fate choice. From Sel. int. DP to Sel. int. CD4 transition, IL7 and IL15 exhibited the highest levels of ligand activity, binding primarily to their respective receptors IL7R and IL2RG on cTEC, thereby activating expression of T cell fate genes *STAT5A* and *LTA* while repressing *TCF7* and *LEF1* (**Figure 6D**; **Supplementary Figure 6G**). From Sel. int. DP to Sel. int. CD8, IL7 and TGFB1 showed the highest ligand activity, engaging IL7R on cTEC and CD4 on DC1, respectively, leading to repression of *TCF7*, *LEF1*, *SOX4* (which controls invariant NKT cell differentiation (56)), and *ID3* (**Figure 6E**; **Supplementary Figure 6H**). From Sel. int. CD4 to CD4^+^ T, TGFB1 displayed the highest ligand activity, primarily interacting with TGFBR2 and APP on DC2, resulting in activation of *IL7R* and *LTA* and repression of *SOX4* and *GATA3* (**Figure 6F** and **Supplementary Figure 6I**). From Sel. int. CD8 to CD8^+^ T, CD80 and PLAT showed elevated ligand activity, binding to CD28 on aDC and ITGB2 on mTECI, respectively, leading to activation of the *CCR7* and cell cycle gene *CCND2*, and repression of *SOX4* and anti-proliferative gene *BTG2* (**Figure 6G**; **Supplementary Figure 6J**). Altogether, these data highlight the pivotal role of thymic stromal cells in orchestrating the lineage bifurcation of CD4^+^ and CD8^+^ T cells.

## Discussion

**Figure 7:**
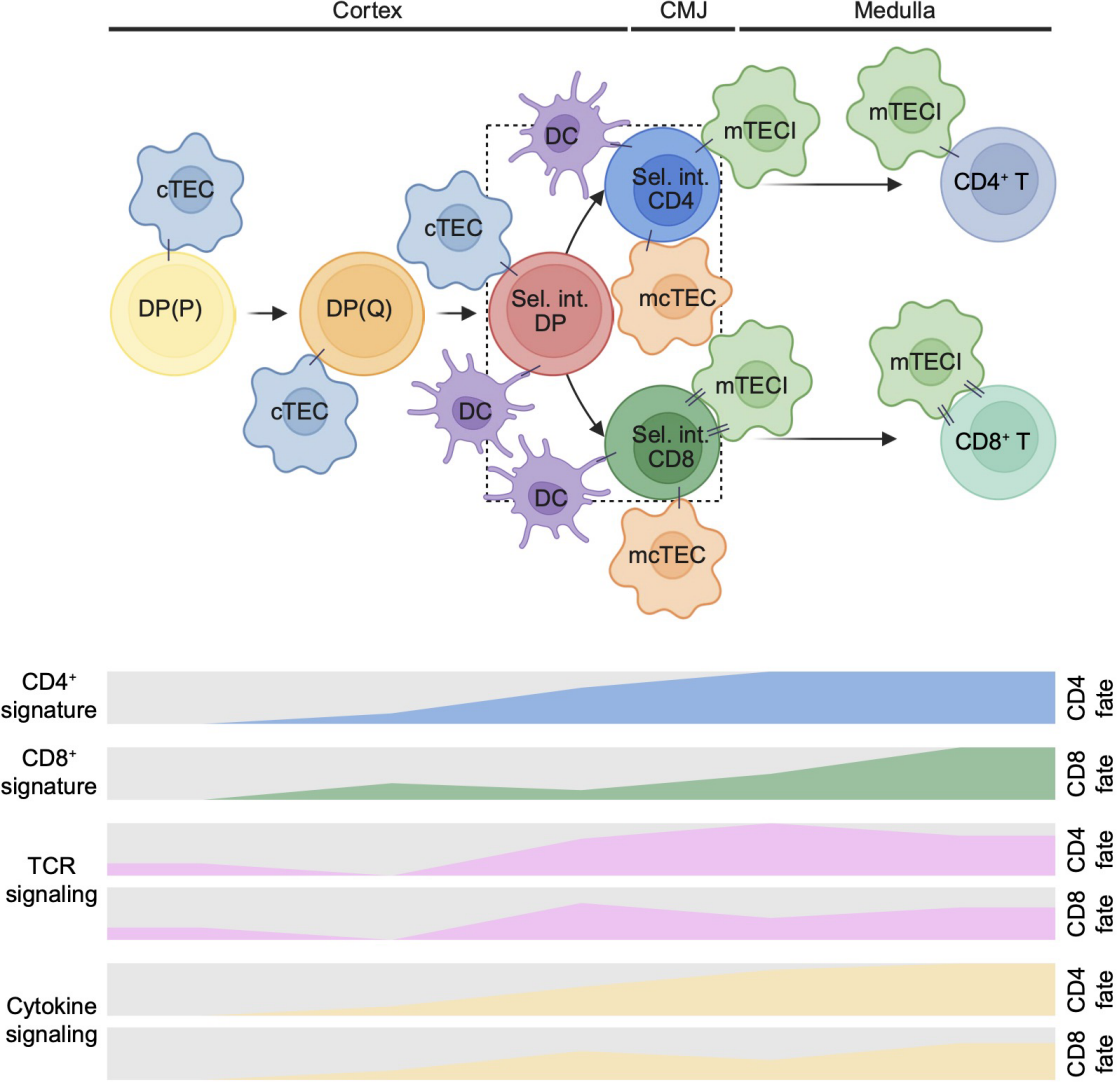
A model for asymmetric CD4/CD8 lineage commitment in the human prenatal thymus. Following positive selection, DP thymocytes differentiate into three distinct selection intermediates, Sel. int. DP, Sel. int. CD4, and Sel. int. CD8. Along the CD4 lineage trajectory, Sel. int. CD4 cells exhibit enriched TCR (indicated by green shading) and cytokine signaling activity (indicated by cyan shading) compared to their CD8 lineage counterparts, reflecting asymmetric signal engagement during lineage specification. CD4 lineage traits (e.g., *CD40LG*, *ICOS*, *ZBTB7B*) emerge as early as Sel. int. CD4 stage, whereas CD8 lineage characteristics (e.g., *NKG7*, *PRF1*, *RUNX3*) only appear at CD8^+^ T cells. Along the CD8 lineage trajectory, Sel. int. CD8 and CD8^+^ T cells display the most extensive interaction networks with mTECIs, implicating enhanced stromal crosstalk in CD8 lineage maturation. Together, these findings reveal that CD4 lineage commitment is characterized by sustained activation signals and robust cytokine responsiveness, while CD8 lineage maturation involves prolonged microenvironmental interactions and delayed lineage gene upregulation. Arrows indicate developmental progression. The numbers of lines connecting thymocytes to stromal cells represent the strength ligand-receptor interactions. CMJ: corticomedullary junction; DP: double-positive; cTEC: cortical thymic epithelial cell; mTEC: medullary thymic epithelial cell; DC: dendritic cell. This figure was created using BioRender (https://biorender.com/).

In this study, we present a comprehensive single-cell atlas of human prenatal thymocytes that captures the cellular heterogeneity and developmental dynamics underlying T cell lineage commitment. Our findings extend previous observations from mouse models (11, 57) and recent human thymus atlases (15–19) by providing a detailed characterization of selection intermediates, transient populations positioned between DP and SP stages that have received limited attention in prior analyses. We uncovered three key features of human thymic selection intermediates (**Figure 7**). First, Sel. int. CD4 cells exhibit enriched TCR and cytokine signaling activity compared to their Sel. int. CD8 counterparts, suggesting asymmetric signal engagement during lineage specification. Second, we observed that CD4 lineage traits emerge coincident with cellular activation, whereas CD8 lineage characteristics appear subsequently, revealing distinct temporal dynamics underlying lineage commitment. Third, Sel. int. CD8 cells and CD8^+^ T cells display the most extensive interaction networks with thymic microenvironmental cells, implicating enhanced stromal crosstalk in CD8 lineage maturation. Together, these findings provide a framework for understanding the cellular and molecular mechanisms governing CD4/CD8 lineage choice in the developing human thymus.

The identification of three distinct selection intermediates (Sel. int. DP, Sel. int. CD4, and Sel. int. CD8) based on coreceptor transcript expression reveals an unappreciated complexity in human thymic development. These populations exhibit downregulation of *RAG1* and *RAG2* alongside upregulation of TCR signaling targets *CD69* and *TOX*, confirming their post-selection status. Notably, the enrichment of AP-1 family regulons (JUN, JUNB, JUND) in Sel. int. CD8 cells aligns with established roles for these transcription factors in T cell differentiation, activation, and proliferation (58). Conversely, BACH2 enrichment in Sel. int. CD4 cells is consistent with its known function in CD4^+^ T cell differentiation (59). The differential regulon activities between CD4^+^ and CD8^+^ T cells largely recapitulate those observed in their respective selection intermediates, suggesting that lineage-specific transcriptional programs are established prior to full maturation. The upregulation of IRF family regulons (IRF4, IRF7) in mature single-positive cells but not in selection intermediates implies that additional waves of transcriptional activation are required to complete lineage specification and prepare for peripheral functions.

The metabolic profiling of selection intermediates reveals T cell maturation involves a dynamic interplay between metabolic rewiring and the progressive acquisition of immune functions. In the early stage, DP (P) cells rely on active acetyl-CoA metabolism to support rapid proliferation (60), while DP (Q) cells shift toward lipid metabolism pathways linked to membrane synthesis and energy remodeling (61), likely preparing for survival and positional shifts within the thymus. The enrichment of glycosaminoglycan biosynthesis in Sel. int. DP cells, specifically heparan sulfate/heparin pathways that serve as coreceptors for fibroblast growth factor and chemokines (41, 42, 62), suggests that these cells are poised to receive and integrate microenvironmental signals, potentially facilitating medullary homing and thymic selection. As cells advance, Sel. int. CD4 cells upregulate branched-chain amino acid, selenocysteine, and retinol metabolism pathways, indicating robust protein biosynthesis and post-translational modification activities, potentially reflecting preparation for effector functions. In contrast, Sel. int. CD8 cells exhibit preferential enrichment of fatty acid and derivative metabolism pathways, which may equip them for rapid cytotoxic responses upon activation, particularly in inflammatory or tumor contexts (63, 64). Interestingly, mature CD4^+^ and CD8^+^ T cells largely converge in their metabolic profiles, implying that lineage-specific metabolic specialization is most critical during the selection intermediate stage, when fate decisions and functional programming occur, and becomes less distinct once cells reach full maturation, possibly reflecting a common baseline metabolic state that supports homeostasis while retaining the capacity for context-dependent activation. Thus, metabolic programs not only fuel but also guide the timing and nature of immune functional acquisition throughout T cell development.

Our finding that CD4 lineage traits emerge coincident with cellular activation while CD8 lineage characteristics follow later provides temporal resolution to longstanding questions about lineage commitment asymmetry. This observation resonates with the “sequential selection” model proposed by Baldwin and Robey (5), wherein differential signal strengths and kinetics dictate lineage outcomes. Our results are also consistent with a single-cell study in mouse thymus that mapped the order and logic of CD4 versus CD8 lineage choice (11). Using MHC II^-^/^-^ mice model, the authors revealed Cd4^+^Cd8a^-^ selection intermediates appear before Cd4^+^Cd8a^-^ selection intermediates, with the timing of these subsets being determined by the availability of MHC class II. More recent work by Steier et al. (12) demonstrated that CD4 lineage differentiation is initiated by an initial wave of TCR signaling, while CD8 lineage specification requires a subsequent, distinct signaling phase. Our human data both resonate with and temporally refine this kinetic model. Specifically, we observed that in human Sel. int. CD4 cells, CD4 lineage genes (*CD40LG*) are expressed coincident with activation markers (*ITM2A*), whereas in the CD8 lineage, upregulation of CD8 effector gene (*NKG7*) occurs only after activation markers have subsided. This suggests that in humans, as in mice, CD4 lineage commitment is coupled to an initial wave of TCR signaling, while CD8 lineage specification is delayed, occurring after the resolution of activation signals. Thus, our data extend the Steier model to human prenatal development and provide the first temporal quantification of this asymmetry in the human thymus. Additionally, studies from the Singer group (13, 65–68) established that the strength and duration of TCR signals, integrated with cytokine cues, are critical for asymmetric lineage outcomes, with CD8 commitment requiring persistent signaling. Collectively, these mouse studies support a model where MHC class-specific signal strength governs the temporal order of selection intermediate emergence. More recently, a study revealed that functionally distinct CD8^+^ T cells are selected by different MHC-I thymic peptides (14), further highlighting the peptide-specific nature of lineage instruction. However, due to the lack of appropriate experimental models in humans, whether this MHC-dictated temporal asymmetry of selection intermediates is conserved in human thymic development remains an open question that warrants future investigation.

Cell-cell communication analyses extend previous observations of thymic crosstalk (69) by systematically mapping interactions between thymocyte subsets and stromal cells. The prominent CCL25−CCR9 and CXCL12−CXCR4 interactions between cTECs and DP cells in the cortex corroborate established roles for these chemokine axes in thymic homing and retention (52). The transition to CCL19−CCR7-mediated interactions with mTECI in selection intermediates aligns with the requirement for medullary migration during negative selection (70). Notably, Sel. int. CD8 cells exhibited the most extensive interaction networks with thymic microenvironmental cells, consistent with recent spatial mapping studies showing that CD8-committed T cells linger longer in the cortex before medullary entry (20).

Limitations of this study. First, the integration of five heterogeneous datasets, while increasing statistical power and temporal coverage, introduces potential confounders. Differences in sequencing platforms, cell sorting strategies, and sampling densities across the original studies may influence clustering, trajectory inference, and the identification of rare populations. Although integration effectively mitigated technical batch effects, biological and experimental variations cannot be fully eliminated. Therefore, future studies using a unified experimental design across the full developmental time course would be valuable to validate our integrated atlas and findings. Second, our conclusions regarding TCR signaling strength, cytokine signaling, and lineage-specific regulon activities are based on inferred pathway activities (e.g., IPA, SCENIC regulon analysis) rather than direct measurements of signaling molecule expression or transcription factor binding. These computational methods provide valuable hypotheses but are inherently indirect. We should interpret these findings as correlational patterns that prioritize candidates for future mechanistic studies. Third, this study is limited by the lack of direct experimental validation of the identified selection intermediate populations. While our single-cell transcriptomic analyses reveal distinct metabolic and signaling programs, these findings remain correlational. Future studies integrating CITE-seq (cellular indexing of transcriptomes and epitopes by sequencing) and other technologies to simultaneously profile surface protein and transcriptomic dynamics, along with functional perturbations in *in vitro* model systems (e.g. thymic organoid systems) or in humanized mouse models, will be essential to experimentally validate the identity, lineage potential, and functional roles of these transient selection intermediates.

In conclusion, this integrative single-cell atlas provides a comprehensive framework for understanding human prenatal T cell development. The identification and characterization of selection intermediates, coupled with detailed analysis of their transcriptional programs, metabolic states, and microenvironmental interactions, advances our understanding of CD4/CD8 lineage commitment in human prenatal thymus. These findings may inform strategies for *in vitro* T cell regeneration and thymus rejuvenation, with potential implications for immunotherapy and treatments of T cell immunodeficiencies.

## Materials and methods

### Data sources

Publicly available scRNA-seq datasets of human embryonic and fetal thymus were collected from five independent studies spanning 7 to 23 post-conception weeks (PCW). Data from Zeng et al. (15) (GSE133341) and Bautista et al. (22) (GSE147520) were downloaded from the Gene Expression Omnibus (GEO). Data from Park et al. (16) (E-MTAB-8581) were obtained from ArrayExpress. Data from Li et al. (23) (OEP001185) were retrieved from the NODE database. Data from Li et al. (24) (HRA007984) were downloaded from the Genome Sequence Archive for Human (GSA-Human). All datasets were generated using 10× Genomics Chromium 3′ or 5′ single-cell RNA-seq. Detailed sample information, including developmental stage, sorting strategy, and sequencing platform, is provided in **Supplementary Table 1**.

### Data processing and quality control

To ensure analytical consistency, all datasets were reprocessed through a unified computational pipeline. Most datasets were obtained as pre-processed count matrices in h5ad format. For datasets in which genes were annotated by Ensembl gene IDs rather than gene symbols, ID-to-symbol conversion was performed using the gene annotation GTF file from the Cell Ranger GRCh38-2020-A reference; when multiple Ensembl IDs mapped to the same gene symbol, the entry with the highest total expression was retained. For a subset of samples from Park et al. (16) for which only aligned BAM files were available, sequencing reads were first recovered using *bamtofastq* (10× Genomics) and then re-aligned to the GRCh38-2020-A reference genome using Cell Ranger v9.0.1 (10× Genomics), thereby generating count matrices under the same reference annotation as all other datasets. The five studies employed different cell enrichment strategies (including CD45^+^ sorting, CD326^+^ sorting, and unsorted thymic cell suspensions; **Supplementary Table 1**); this heterogeneity was deliberately retained to capture both hematopoietic and non-hematopoietic thymic populations and was accounted for during data integration.

Uniform quality control criteria were applied to all cells across all datasets. Cells were retained if they expressed between 500 and 5,000 genes, contained between 1,000 and 20,000 unique molecular identifiers (UMIs), and had a mitochondrial read fraction of 10% or less. Doublets were identified and removed independently for each sample using Scrublet, as implemented in Scanpy (*sc.pp.scrublet*), with the expected doublet rate estimated automatically based on the number of recovered cells per sample. After filtering, 216,463 cells spanning 48,903 genes were retained for downstream analysis (median: 1,635 genes per cell, mean: 1,868; median: 4,326 UMIs per cell, mean: 5,879; per-dataset UMI distributions are shown in **Supplementary Figure 1A**). Detailed sample information, including developmental stage, sorting strategy, sequencing platform, and pre- and post-quality-control cell numbers, is provided in **Supplementary Table 1**. T lineage cells (84,025 cells) and thymic microenvironment (TME) cells (15,750 cells) were then analyzed separately, and each subset was further filtered to retain cells with more than 1,000 detected genes.

### Data integration, clustering, and batch effects correction

Data integration and clustering were performed using Scanpy v1.9.1 (71). Raw UMI counts were normalized to a total of 10,000 counts per cell and log-transformed (log1p). A total of 5,000 highly variable genes (HVGs) were selected using the *highly_variable_genes* function with the batch key set to dataset of origin. Following mean-centering and unit-variance scaling, principal component analysis (PCA) was performed on the HVG expression matrix, and the top 50 principal components were retained.

Batch effects arising from differences in study of origin and sequencing platform (10× Chromium 3′ and 5′) were corrected using BBKNN (batch balanced k-nearest neighbours) (72), with the study of origin designated as the batch variable. Unsupervised clustering was performed on the batch-corrected neighborhood graph using the Leiden algorithm (igraph implementation, n_iterations = 2, directed = False) at a resolution of 4.5, yielding 102 initial clusters. Cells were visualized in two dimensions using uniform manifold approximation and projection (UMAP). Batch correction was assessed by visual inspection of UMAP embeddings colored by dataset of origin (**Supplementary Figure 1B**), sequencing platform (**Supplementary Figure 1C**), sorting strategy (**Supplementary Figure 1D**), and developmental stage (**Supplementary Figure 1E**). Cell type annotation was performed using CellTypist (73) as an initial automated reference, followed by systematic manual curation in which the 102 Leiden clusters were evaluated based on marker gene expression profiles and merged into biologically coherent populations. A total of 22 cell populations were defined, comprising 13 T lineage populations (ETP, DN-early, DN(P), DN(Q), DP(P), DP(Q), αβ T(entry), CD4^+^ T, CD8^+^ T, Treg, CD8αα, NKT, and γδ T) and 9 non-T populations (B, Endothelial, Epithelial, Erythroid, Fibroblast, Mesothelium, Myeloid, Schwann, and VSMC).

### Sub-clustering analysis

To examine cellular heterogeneity within major compartments, iterative sub-clustering was performed. For the αβ T(entry) population, differentially expressed genes were used as features for PCA, and three subsets (Sel. int. DP, Sel. int. CD4, and Sel. int. CD8) were manually annotated based on canonical marker expression. For the epithelial, myeloid, and dendritic cell compartments, HVG-based PCA and BBKNN integration were applied independently, followed by UMAP recalculation for visualization. Cell type annotation was performed using CellTypist (73). Epithelial cells were divided into nine TEC subsets, myeloid cells into five subsets, and dendritic cells into four subtypes (DC1, DC2, aDC, and pDC).

### Differential expression analysis

Differentially expressed genes (DEGs) were identified using the Wilcoxon rank-sum test implemented in Scanpy (71), with significance defined as adjusted *P* < 0.05 (Benjamini-Hochberg correction). To define CD4^+^ and CD8^+^ lineage-specific gene signatures, DEGs were evaluated at both the αβ T(entry) and mature SP stages. For CD4^+^ signatures, genes were required to have log_2_FC > 1.0 relative to the DP stage and log_2_FC > 0.8 relative to the CD8^+^ lineage at both stages, with FDR < 0.05 at both stages. For CD8^+^ signatures, relaxed entry-stage criteria were applied, as lineage commitment markers are more pronounced at the mature stage: log_2_FC > 1.0 relative to the DP stage, log_2_FC > 0.4 relative to the CD4^+^ lineage at the entry stage and log_2_FC > 0.6 at the mature stage, with FDR < 0.05 at the mature stage. This analysis yielded 51 CD4^+^ and 37 CD8^+^ lineage signature genes (**Supplementary Table 2**).

### Gene set scoring

Gene set activity scores were calculated using *scanpy.tl.score_genes* with curated gene sets derived from Chopp et al. (25).

### Gene regulatory network inference

Gene regulatory networks were inferred using pySCENIC v0.12.1 (74). The workflow consisted of three steps: (1) GRN inference via GRNBoost2 to identify co-expression modules; (2) regulon prediction using cisTarget with *hg38* motif databases to retain only modules with significant *cis*-regulatory enrichment; and (3) cellular regulon activity scoring by AUCell. AUCell scores were Z-score normalized across cell types to generate regulon activity heatmaps, and the top 10 regulons per cell type were selected by statistical significance. Regulon specificity scores (RSS) were calculated using Jensen-Shannon divergence to identify cell type-specific regulons and were visualized as both RSS heatmaps and RSS rank plots.

### Functional enrichment analysis

Gene Ontology (GO) biological process enrichment analysis was performed using the clusterProfiler R package (75) with the org.Hs.eg.db annotation database. DEGs for each cluster were identified by FindAllMarkers (Seurat (76), Wilcoxon rank-sum test, log_2_FC > 0.25, adjusted *P* < 0.05), excluding mitochondrial and ribosomal genes. Redundant GO terms were removed using the *simplify* function (cutoff = 0.7). The top five enriched terms per cluster were visualized.

### Metabolic pathway analysis

Single-cell metabolic pathway activity was scored using scMetabolism (77) with the AUCell method and the KEGG pathway database. Mean pathway activity scores were computed per cell type and Z-score normalized across cell types. The top five metabolic pathways per cell type were selected for visualization.

### Pseudotime and trajectory analysis

Pseudotime trajectories of T-lineage cells were reconstructed using three independent approaches, all with root cells assigned to the DP(P) population. Slingshot (78) (R, default parameters) was used as the primary pseudotime method. Diffusion pseudotime (DPT) was computed in Scanpy, and a transition probability matrix was derived using the PseudotimeKernel in CellRank (79). Monocle 3 (80) (R) was also applied for independent validation. Genes with significant temporal variation along pseudotime were identified using the associationTest function in tradeSeq (81) (FDR < 1e-10). These genes were then clustered using Mfuzz soft clustering, with the optimal number of clusters (k = 4) determined by the gap statistic. GO enrichment analysis of each cluster was performed using ClusterGVis (https://github.com/junjunlab/ClusterGVis).

### Cell-cell communication analysis

#### CellPhoneDB analysis

Ligand-receptor interactions between TME and T-lineage cells were inferred using CellPhoneDB v5 (82) with the statistical analysis mode (1,000 permutations, default parameters). Three anatomical regions were analyzed separately: cortex (cTEC and Macrophage as senders; DP(P), DP(Q), and Sel. int. DP as receivers), cortico-medullary junction (CMJ; mcTEC as sender; αβ T(entry) subsets as receivers), and medulla (mTEC subsets, DC subsets, and B cells as senders; Sel. int. CD4, Sel. int. CD8, CD4^+^ T, and CD8^+^ T as receivers). Significant interactions (*P* < 0.05) were summarized as interaction counts, pathway-level patterns, and chemokine ligand-receptor dot plots.

#### NicheNet analysis

Ligand–target regulatory predictions were performed using the NicheNet R package (83) for four pairwise comparisons of T-lineage receiver cells: Sel. int. DP versus Sel. int. CD4, Sel. int. DP versus Sel. int. CD8, Sel. int. CD4 versus CD4^+^ T, and Sel. int. versus CD8^+^ T. TME cells served as the ligand-expressing sender population, assigned by anatomical region (CMJ for lineage commitment comparisons; medulla for maturation comparisons). DEGs in the receiver population (∣log_2_FC∣ ≥ 0.25, adjusted *P* ≤ 0.01) defined the gene set of interest. Ligand activities were ranked by the area under the precision-recall curve (AUPR). The top non-chemokine ligands, along with their predicted target genes and cognate receptors, were visualized.

### Upstream analysis

DEGs identified by the FindMarkers function in Seurat (Wilcoxon rank-sum test) were subjected to upstream regulator analysis using Ingenuity Pathway Analysis (IPA, QIAGEN) (84) to predict potential upstream transcriptional regulators and their activation states.

### Quantification and statistical analysis

Statistical tests used in each analysis are specified in the corresponding method sections. Unless otherwise stated, the Wilcoxon rank-sum test was used for differential expression analysis, and *P* values were adjusted for multiple testing using the Benjamini-Hochberg method. An adjusted *P* < 0.05 was considered statistically significant. Significance in the CellPhoneDB analysis was determined by permutation testing (1,000 iterations, *P* < 0.05). Ligand activities in the NicheNet analysis were evaluated by AUPR. Upstream regulators identified by IPA were considered active or inhibited when the activation Z-score exceeded 2 or fell below -2, respectively. All analyses were performed using Python (v3.9) and R (v4.2), with specific package versions detailed above.

## Data Availability

The datasets presented in this study can be found in online repositories. The names of the repository/repositories and accession number(s) can be found in the article/**Supplementary Material**.

## Supplementary material

Supplementary Figure 1 An integrative atlas of human prenatal thymocytes. **(A)** Violin plot showing the UMI counts per cell of five datasets. **(B)** Visualization of UMAP for cellular composition of human prenatal thymus colored by data source. **(C)** Visualization of UMAP for cellular composition of human prenatal thymus colored by platform. **(D)** Visualization of UMAP for cellular composition of human prenatal thymus colored by sort strategy. **(E)** Visualization of UMAP for cellular composition of human prenatal thymus colored by gestational stage. **(F)** Dot plot showing feature genes expression in indicated cell clusters. **(G)** Bar graph showing the proportion representation of cell states colored by data source.

Supplementary Figure 2 Identification of selection intermediates. **(A)** Visualization of UMAP for cellular composition of human prenatal thymus colored by cell type. **(B)** Scatter plot showing *CD4* and *CD8A* expression in αβ T(entry) cluster. **(C)** Bar plots showing the proportion (left panel) and numbers (right panel) of indicated cell clusters in five source datasets. **(D)** Bar plots showing the composition of indicated cell clusters across different stages. **(E)** For each indicated cell population, the proportion contributed by different ages is shown as a bar plot. **(F)** Violin plots show cluster-based average expression scores for TCR signal-induced genes (left), CD4^+^-lineage (middle), and CD8^+^-lineage signature genes (right).

Supplementary Figure 3 Enriched regulons activities in distinct clusters. **(A)** Top 10 enriched regulons activities were shown in Sel. int. DP cluster. **(B)** Top 10 enriched regulons activities were shown in Sel. int. CD4 cluster. **(C)** Top 10 enriched regulons activities were shown in Sel. int. CD8 cluster. **(D)** Top 10 enriched regulons activities were shown in CD4^+^ T cluster. **(E)** Top 10 enriched regulons activities were shown in CD8^+^ T cluster.

Supplementary Figure 4 Expression of genes related to cytokine signaling and pathways by selection intermediates **(A)** Expression of indicated interested genes by selection intermediates. **(B)** IPA analysis of pathway activity downstream of the indicated cytokines and cytokine signaling molecules. Activation Z-scores above 2 and below -2 are considered significant.

Supplementary Figure 5 Developmental trajectory analysis. **(A)** Developmental trajectory of thymocytes inferred by RNA velocity. **(B)** Developmental trajectory of thymocytes inferred by Monocle 3. **(C)** Developmental trajectory of thymocytes inferred by Monocle 3 pseudotime.

Supplementary Figure 6 Stromal cell re-clustering and cell-cell communication analysis. **(A)** Visualization of UMAP for cellular composition of myeloid cells colored by cell type. **(B)** Dot plot showing feature genes expression in indicated myeloid cell clusters. **(C)** Visualization of UMAP for cellular composition of DC cells colored by cell type. **(D)** Dot plot showing feature genes expression in indicated DC cell clusters. **(E)** Visualization of UMAP for cellular composition of TECs colored by cell type. **(F)** Dot plot showing feature genes expression in indicated TECs clusters. **(G)** The ligand activity is shown from Sel. int. DP to Sel. int. CD4. **(H)** The ligand activity is shown from Sel. int. DP to Sel. int. CD8. **(I)** The ligand activity is shown from Sel. int. CD4 to CD4^+^ T. **(J)** The ligand activity is shown from Sel. int. CD8 to CD8^+^ T.
