# Supplementary figures and images for: Single-cell transcriptome delineating asymmetric dynamics of CD4+ T and CD8+ T cell lineage commitment in human prenatal thymus
Source: Front Immunol. 2026 May 19;17:1823728. doi: 10.3389/fimmu.2026.1823728 (PMC13226484; doi:10.3389/fimmu.2026.1823728)

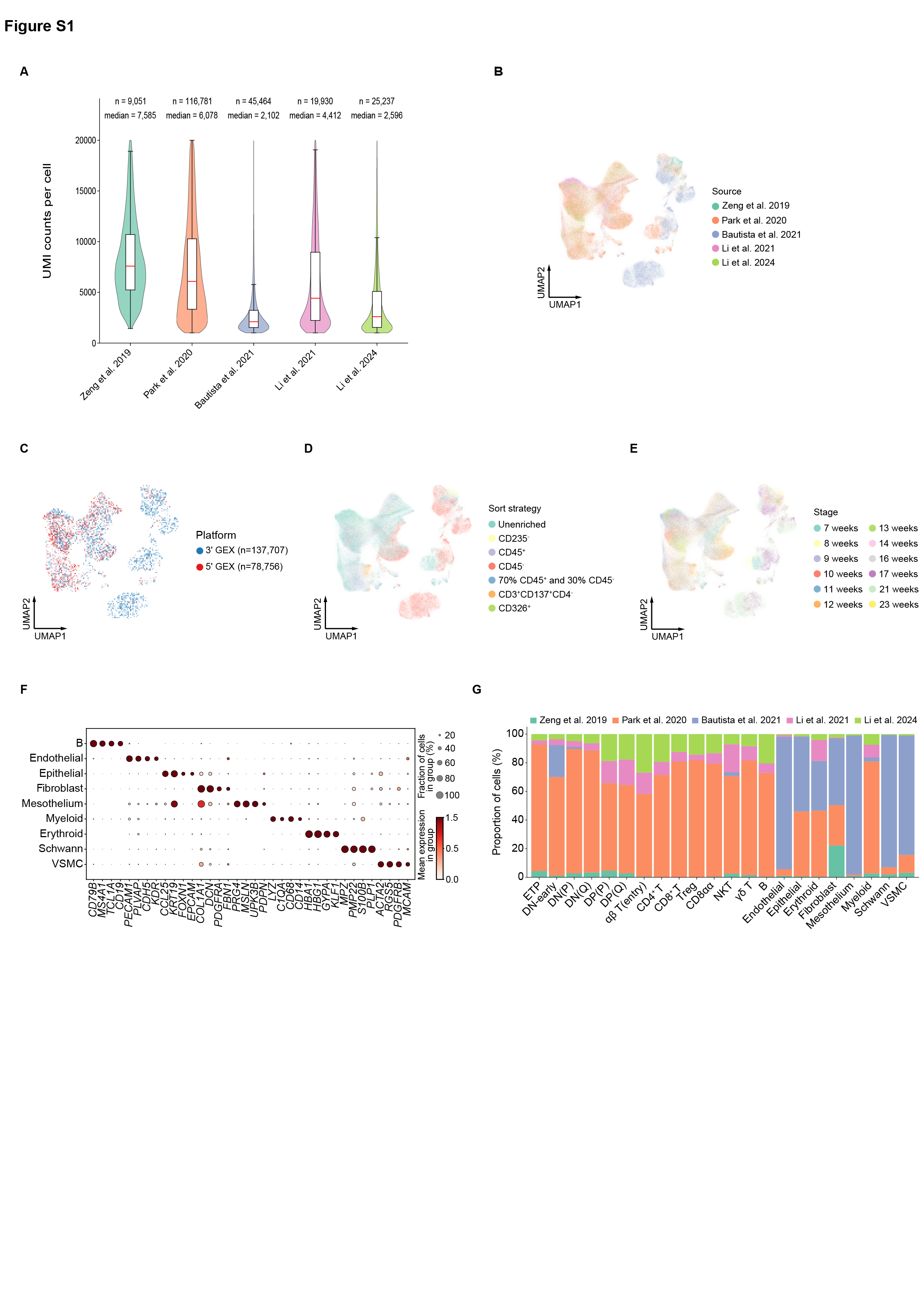

Supplement: Supplementary Figure 1 — An integrative atlas of human prenatal thymocytes. (A) Violin plot showing the UMI counts per cell of five datasets. (B) Visualization of UMAP for cellular composition of human prenatal thymus colored by data source. (C) Visualization of UMAP for cellular composition of human prenatal thymus colored by platform. (D) Visualization of UMAP for cellular composition of human prenatal thymus colored by sort strategy. (E) Visualization of UMAP for cellular composition of human prenatal thymus colored by gestational stage. (F) Dot plot showing feature genes expression in indicated cell clusters. (G) Bar graph showing the proportion representation of cell states colored by data source. [file Image1.jpeg]

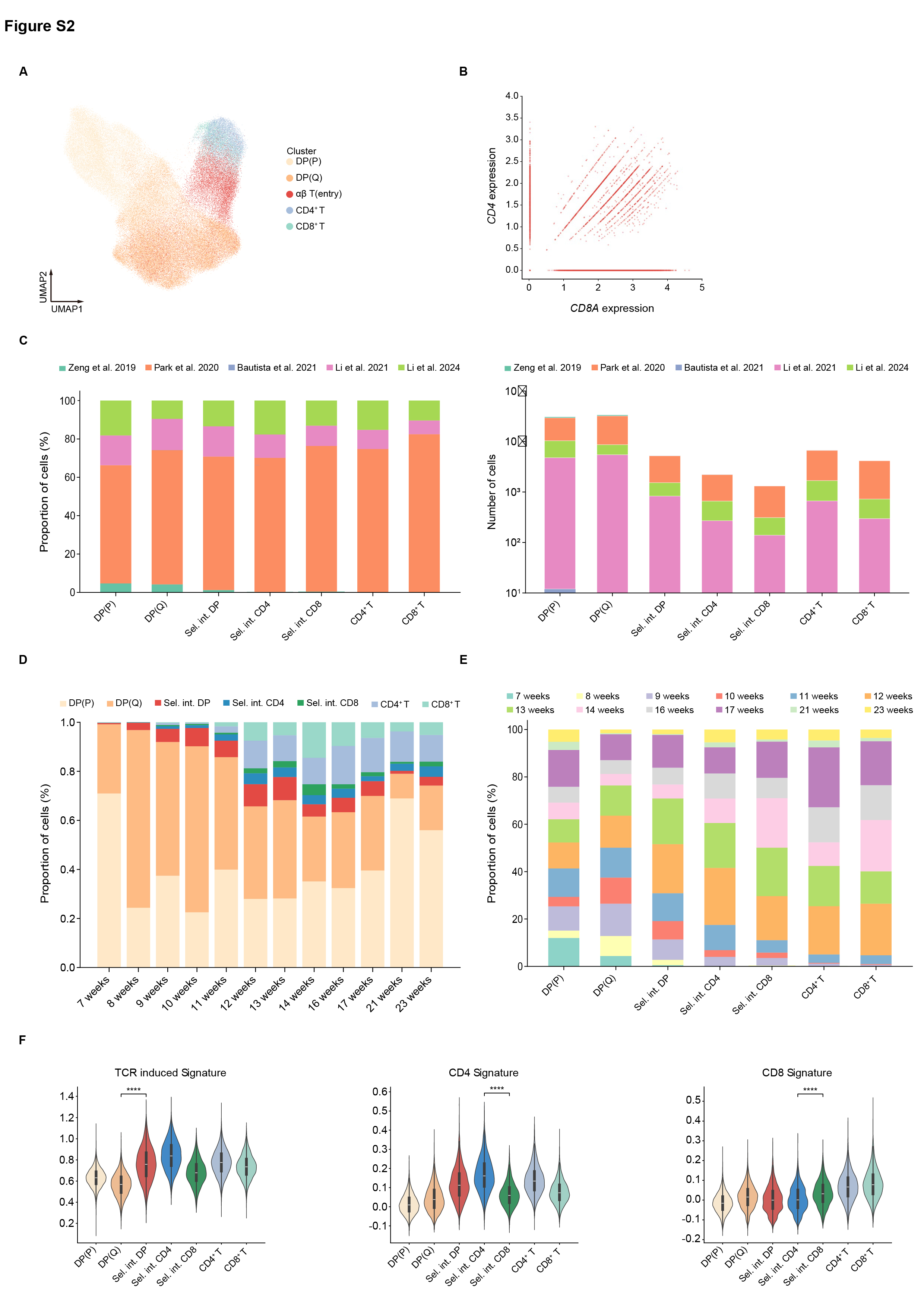

Supplement: Supplementary Figure 2 — Identification of selection intermediates. (A) Visualization of UMAP for cellular composition of human prenatal thymus colored by cell type. (B) Scatter plot showing CD4 and CD8A expression in αβ T(entry) cluster. (C) Bar plots showing the proportion (left panel) and numbers (right panel) of indicated cell clusters in five source datasets. (D) Bar plots showing the composition of indicated cell clusters across different stages. (E) For each indicated cell population, the proportion contributed by different ages is shown as a bar plot. (F) Violin plots show cluster-based average expression scores for TCR signal-induced genes (left), CD4+-lineage (middle), and CD8+-lineage signature genes (right). [file Image2.jpeg]

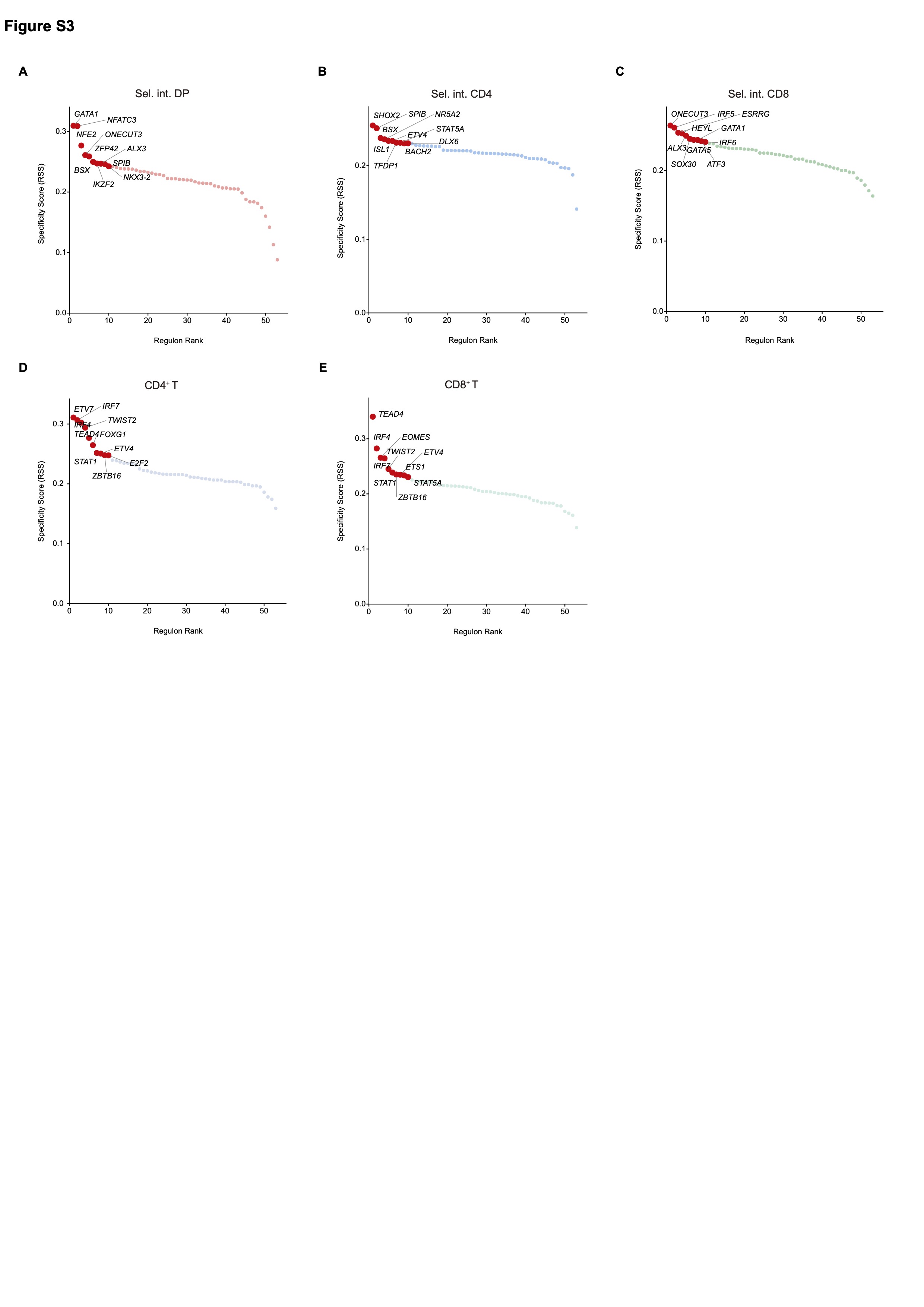

Supplement: Supplementary Figure 3 — Enriched regulons activities in distinct clusters. (A) Top 10 enriched regulons activities were shown in Sel. int. DP cluster. (B) Top 10 enriched regulons activities were shown in Sel. int. CD4 cluster. (C) Top 10 enriched regulons activities were shown in Sel. int. CD8 cluster. (D) Top 10 enriched regulons activities were shown in CD4+ T cluster. (E) Top 10 enriched regulons activities were shown in CD8+ T cluster. [file Image3.jpeg]

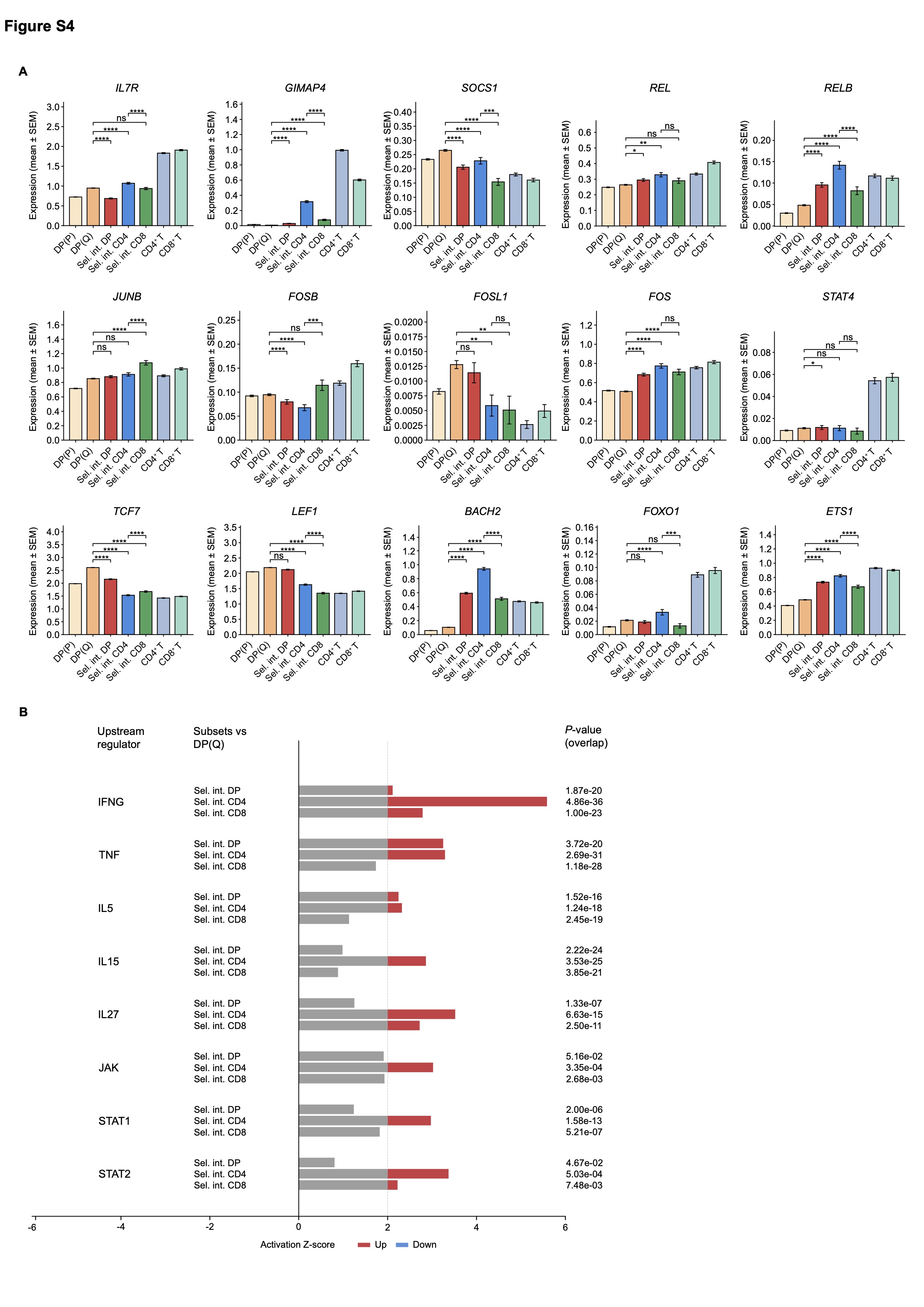

Supplement: Supplementary Figure 4 — Expression of genes related to cytokine signaling and pathways by selection intermediates (A) Expression of indicated interested genes by selection intermediates. (B) IPA analysis of pathway activity downstream of the indicated cytokines and cytokine signaling molecules. Activation Z-scores above 2 and below -2 are considered significant. [file Image4.jpeg]

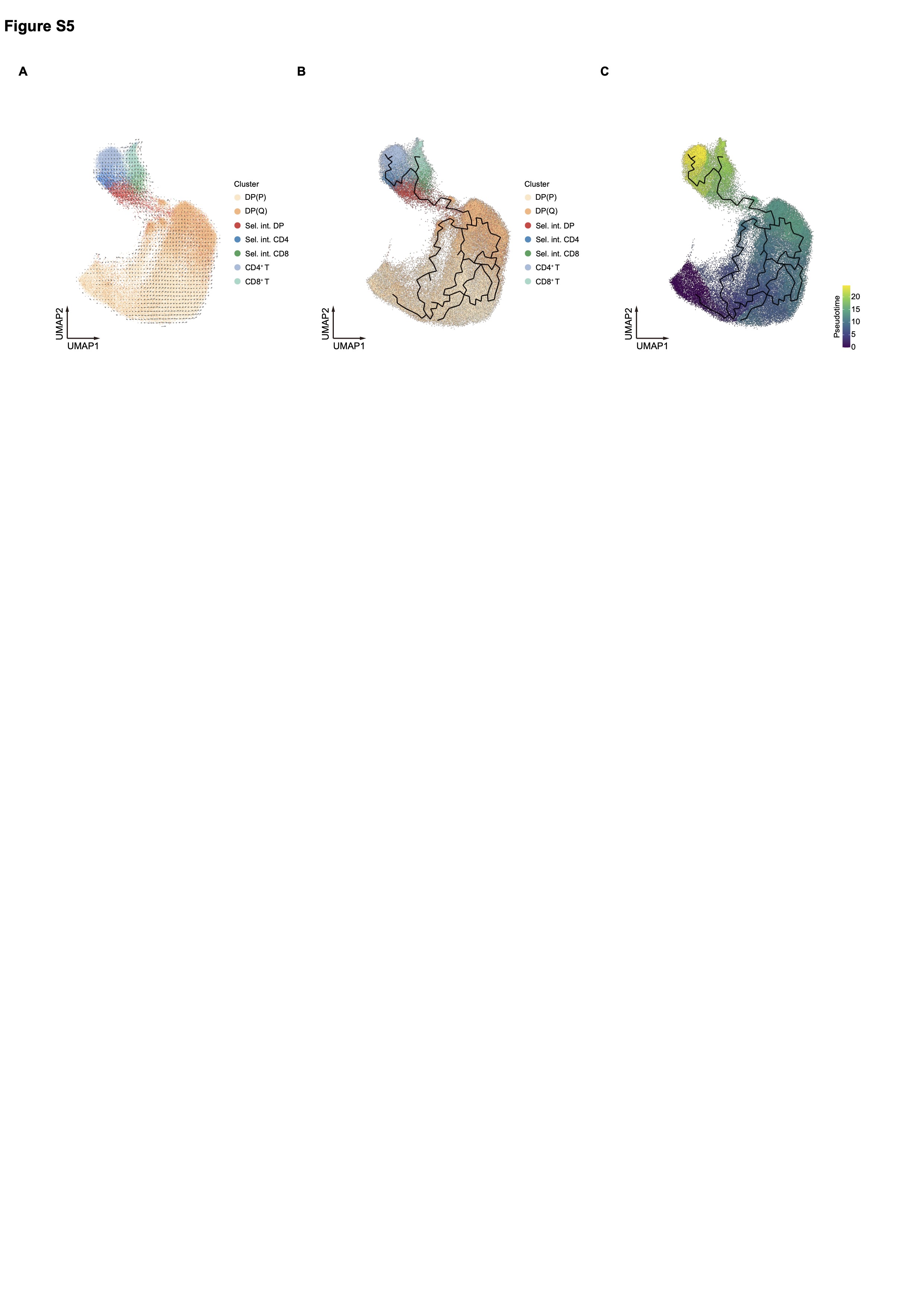

Supplement: Supplementary Figure 5 — Developmental trajectory analysis. (A) Developmental trajectory of thymocytes inferred by RNA velocity. (B) Developmental trajectory of thymocytes inferred by Monocle 3. (C) Developmental trajectory of thymocytes inferred by Monocle 3 pseudotime. [file Image5.jpeg]

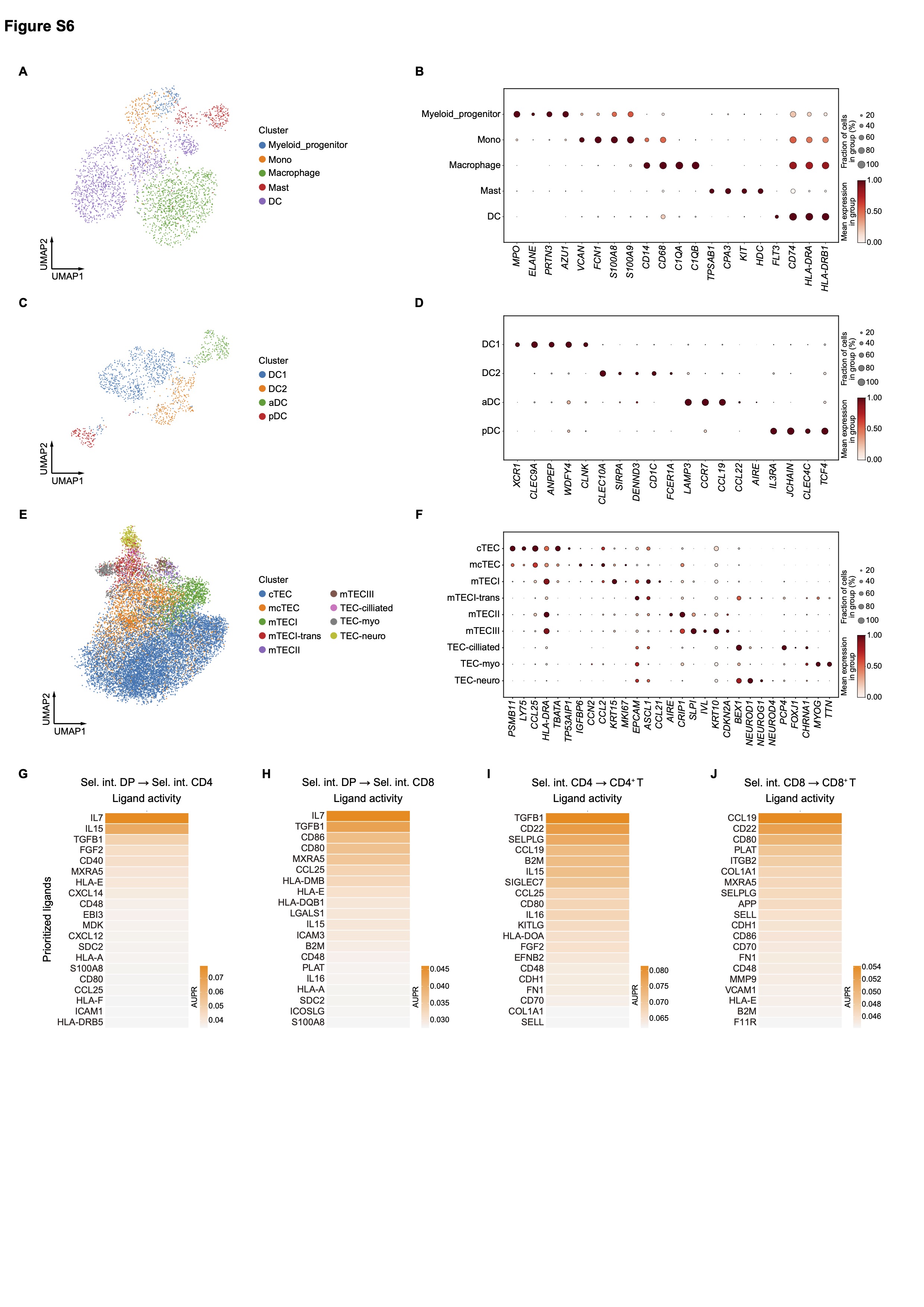

Supplement: Supplementary Figure 6 — Stromal cell re-clustering and cell-cell communication analysis. (A) Visualization of UMAP for cellular composition of myeloid cells colored by cell type. (B) Dot plot showing feature genes expression in indicated myeloid cell clusters. (C) Visualization of UMAP for cellular composition of DC cells colored by cell type. (D) Dot plot showing feature genes expression in indicated DC cell clusters. (E) Visualization of UMAP for cellular composition of TECs colored by cell type. (F) Dot plot showing feature genes expression in indicated TECs clusters. (G) The ligand activity is shown from Sel. int. DP to Sel. int. CD4. (H) The ligand activity is shown from Sel. int. DP to Sel. int. CD8. (I) The ligand activity is shown from Sel. int. CD4 to CD4+ T. (J) The ligand activity is shown from Sel. int. CD8 to CD8+ T. [file Image6.jpeg]
